# Identification and validation of aging-related genes in atrial fibrillation

**DOI:** 10.1371/journal.pone.0294282

**Published:** 2023-11-13

**Authors:** Yong Zhou, Chao Sun, Yingxu Ma, Yunyin Huang, Keke Wu, Shengyuan Huang, Qiuzhen Lin, Jiayi Zhu, Zuodong Ning, Ningyuan Liu, Tao Tu, Qiming Liu

**Affiliations:** 1 Department of Cardiovascular Medicine, The Second Xiangya Hospital of Central South University, Changsha City, Hunan Province, China; 2 Department of Cardiovascular Surgery, The Second Xiangya Hospital of Central South University, Changsha City, Hunan Province, China; 3 Xiangya School of Medicine, Central South University, Changsha, Hunan, China; Dasman Diabetes Institute, KUWAIT

## Abstract

Atrial fibrillation (AF) is the most common sustained cardiac arrhythmia in the clinic. Aging plays an essential role in the occurrence and development of AF. Herein, we aimed to identify the aging-related genes associated with AF using bioinformatics analysis. Transcriptome profiles of AF were obtained from the GEO database. Differential expression analysis was performed to identify AF-specific aging-related genes. GO and KEGG enrichment analyses were performed. Subsequently, the LASSO, SVM-RFE, and MCC algorithms were applied to screen aging-related genes. The mRNA expression of the screened genes was validated in the left atrial samples of aged rapid atrial pacing-induced AF canine models and their counterparts. The ROC curves of them were drawn to evaluate their diagnostic potential. Moreover, CIBERSORT was used to estimate immune infiltration. A correlation analysis between screened aging-related genes and infiltrating immune cells was performed. A total of 24 aging-related genes were identified, which were found to be mainly involved in the FoxO signaling pathway, PI3K-Akt signaling pathway, longevity regulating pathway, and peroxisome according to functional enrichment analysis. LASSO, SVM-RFE, and MCC algorithms identified three genes (*HSPA9*, *SOD2*, *TXN*). Furthermore, the expression levels of *HSPA9* and *SOD2* were validated in aged rapid atrial pacing-induced AF canine models. *HSPA9* and *SOD2* could be potential diagnostic biomarkers for AF, as evidenced by the ROC curves. Immune infiltration and correlation analysis revealed that *HSPA9* and *SOD2* were related to immune cell infiltrates. Collectively, these findings provide novel insights into the potential aging-related genes associated with AF. *HSPA9* and *SOD2* may play a significant role in the occurrence and development of AF.

## Introduction

Atrial fibrillation (AF) is the most common sustained cardiac arrhythmia and is associated with increased risk of stroke, heart failure, and cardioembolic hospitalization [1, 2]. AF is a multifaceted and heterogeneous disease, with numerous epidemiological studies unambiguously showcasing that aging constitutes the foremost risk factor for AF development [3]. The incidence of AF escalates significantly with age, as substantiated by a Scottish study, which documented first ever incidence rates of 1.1%, 3.2%, 6.2%, and 7.7% for individuals aged 55–64, 65–74, 75–84, and ≥85 years, correspondingly [4].

Aging is a complex physiological and pathophysiological process that triggers senescence, oxidative stress, and inflammation, all of which play a crucial role in the progression of AF [5]. Analyses of blood samples have suggested that AF patients exhibit shortened telomeres in comparison to those in sinus rhythm (SR) [6]. Furthermore, translational profiling of atrial appendages has unveiled that paroxysmal/persistent AF patients, aged between 58 and 82 years, manifest heightened senescence and proinflammatory biomarkers in contrast to SR cohorts aged between 57 and 79 years [7]. Additionally, the expressions of p53 and p16 increased, and the expression of endothelial nitric oxide synthase (eNOS) declined in the right atrial appendages of AF patients [7]. At present, atrial remodeling, encompassing structural and electrical remodeling, is widely recognized as a pivotal factor in the pathogenesis of AF [8]. Notably, atrial fibrosis has emerged as a significant pathophysiological contributor, demonstrating associations with AF recurrences [9]. Multiple studies suggest underscore senescence as a key contributor to AF, may through the induction of atrial fibrosis mediated by extracellular matrix, angiotensin II, TGF-β/Smad, and thrombin signaling pathways [5, 10–12]. However, it remains uncertain whether aging-related genes play a pivotal role in the development of AF. Thus, investigating the relationship between aging-related genes and AF holds the potential to unveil novel therapeutic targets for this disease.

Accumulating evidence has demonstrated the involvement of immune cells in the pathogenesis of AF [13, 14]. Elevated levels of pro-inflammatory cytokines, including C-reactive protein (CRP), interleukin-6 (IL-6), and tumor necrosis factor-α (TNF-α), have been linked to the progression of AF in patients [15, 16]. Prior research suggests that cellular senescence may impact the pathology of various diseases through the secretion of inflammatory chemokines, immune modulators, and other factors [5]. Furthermore, substantial evidence indicates that the inability to finely control systemic inflammatory responses may serve as a marker of unsuccessful aging [17]. Whether the aging-associated immune response also play a role aging-related AF remains unknown. Therefore, our study aimed to comprehensively analyze aging-related genes and their association with immune infiltration in AF using bioinformatic methods.

## Materials and methods

### Data acquisition

**Fig 1:**
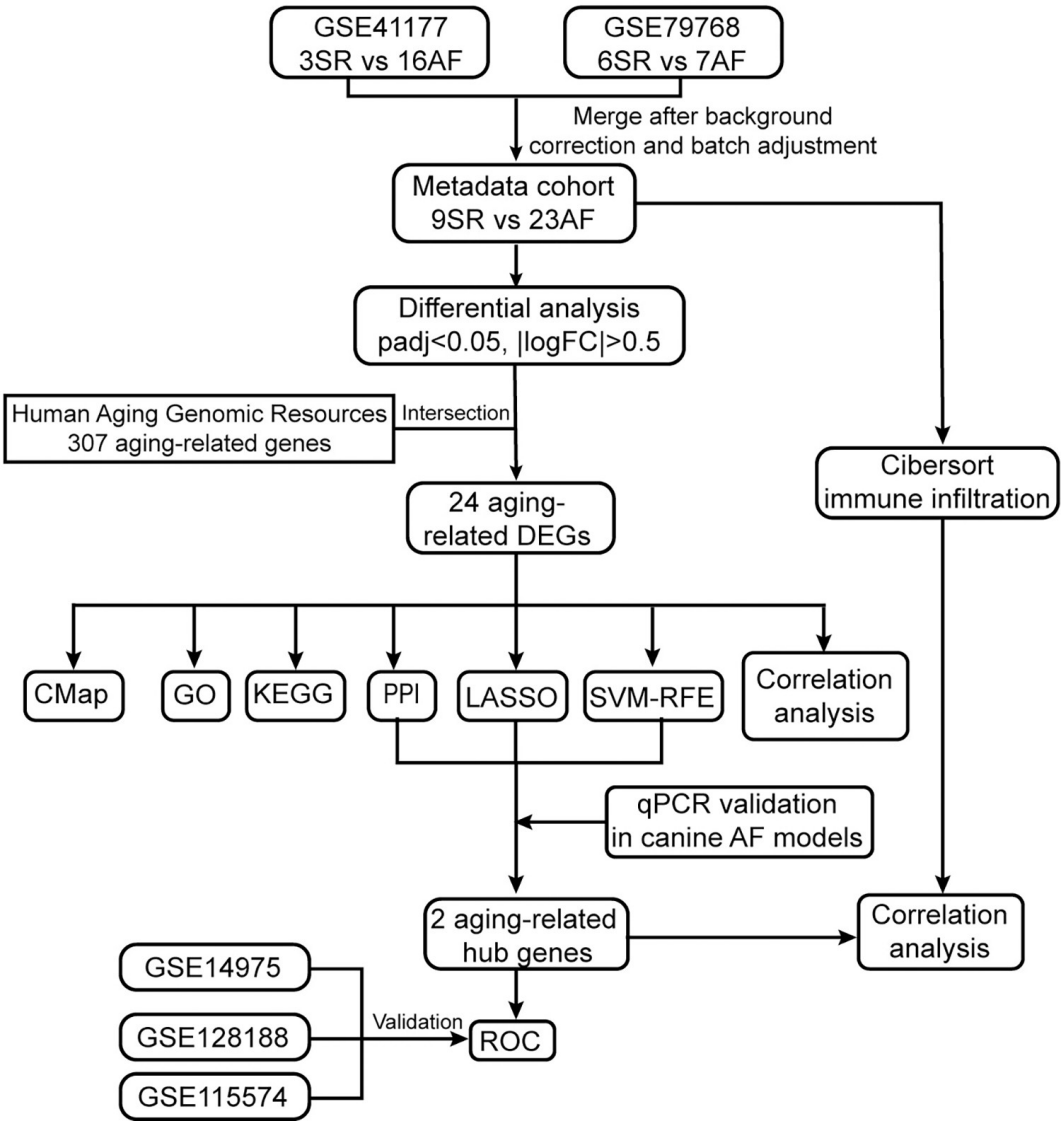
The workflow of this study.

The workflow of this study was presented in **Fig 1**. Transcriptome data generated from AF patients and controls were downloaded from the Gene Expression Omnibus (GEO) repository (https://www.ncbi.nlm.nih.gov/geo/). The GSE79768 and GSE41177 datasets, based on the GPL570 platform, were downloaded. In each dataset, only left atrial samples from AF and SR patients were retrieved. The GSE79768 dataset contained 13 left atrial tissue samples from 7 AF patients and 6 SR individuals [18]. The GSE41177 dataset contained 19 left atrial tissue samples from 16 AF patients and 3 SR patients [19]. The patients’ basic information of GSE79768 and GSE41177 was also obtained from the GEO database and was shown in **S1 Table in S1 File**. The probes were converted to gene symbols according to the probe annotation files. Moreover, the GSE14975, GSE115574, and GSE128188 databases were also downloaded from the GEO database and were used for validation. In this study, 307 aging-related genes were retrieved from the human Aging Genomic Resources [20] (https://genomics.senescence.info/). The list of 307 aging-related genes was shown in **S2 Table in S2 File.**

### Differential expression analysis

Each dataset was background corrected and normalized by the “limma” package. The average of the probes would be calculated as the expression level of the gene when one gene symbol matched more than one probe. GSE79768 and GSE41177 were merged as a metadata cohort for further analysis. The batch effects were eliminated through the combat function of the “SVA” package [21]. The “limma” package was applied to identify differentially expressed genes (DEGs) between AF and SR patients. Genes with adjusted *P* value <0.05 and |log2 FC| >0.5 were considered DEGs. Jvenn [22] was applied to obtain the overlapped genes between DEGs and 307 aging-related genes. The overlapped genes were considered AF aging-related DEGs. The “ggplot2” package were applied to create volcano maps and box plots. The Wilcoxon rank sum test was used to analyze the significance of aging-related DEGs.

### Prediction of small molecular drugs

Aging-related DEGs were input into the Connectivity Map (CMap) database to explore small molecular drugs with the potential to inhibit AF occurrence and development. Subsequently, potential therapeutic compounds were referred to as drugs with negative Raw_cs and high fdr_q_nlog10 values. These compounds were significantly negatively correlated with aging-related DEGs.

### Functional enrichment analysis

Gene Ontology (GO) and Kyoto Encyclopedia of Genes and Genomes (KEGG) pathway enrichment analysis were carried out by the “clusterProfiler” package [23]. The biological process, cell component, and molecular function of aging-related DEGs were separately investigated. Reactome pathway enrichment analysis was performed through “ReactomePA” package [24]. Additionally, Biocarta pathway enrichment analysis was conducted using EnrichR online tool (https://maayanlab.cloud/Enrichr/). The functional enrichment analysis was performed by calculating the hypergeometric distribution relationship between the differential genes and specific branches of the pathway classification. Enrichment analysis will return an assumed p-value for each term in which the differential genes exist. A small p-value indicates the differential genes enriched in specific terms. The *P* value <0.05 was deemed significantly enriched.

### Protein-protein interaction (PPI) and correlation analyses

PPI network of aging-related DEGs was constructed by the Search Tool for the Retrieval of Interacting Genes (STRING database, v11.5, https://string-db.org/) to investigate protein functional association with the interaction score >0.4. The result was downloaded from the STRING database and then visualized by Cytoscape software (version 3.9.1). In the network, nodes represent proteins, and edges represent protein-protein interactions. A Cytoscape’s plugin, cytohubba, was used to investigate the hub genes by Maximal Clique Centrality (MCC) algorithm. MCC identifies central nodes in a network by finding all the maximal cliques, determining how each node participates in these cliques, and assigning centrality scores based on this participation [25]. The top 10 genes were considered hub genes. The “corrplot” R package was applied to perform correlation analysis and visualization of aging-related DEGs. Pearson correlation analysis was used to analyze the correlation among aging-related DEGs.

### Feature selection of aging-related DEGs

Two machine learning algorithms, the least absolute shrinkage and selection operator (LASSO) and support vector machine recursive elimination (SVM-RFE), were used to further screen aging-related genes in AF. LASSO includes the dual features of subset selection and ridge regression and represents a regression analysis algorithm applying regularization for feature selection. LASSO analysis was conducted by “glmnet” package with the turning/penalty parameter utilizing 10-fold cross-validation [26]. The genes were selected based on the optimal lambda value in LASSO analysis. Moreover, some regression coefficients of genes were reduced to zero and excluded. Only the genes with non-zero regression coefficients remained and were considered characteristic genes. SVM-RFE represents a supervised machine learning technique for classification and regression [27]. SVM-RFE was performed to obtain the optimal variables via 10-fold cross-validation utilizing the “caret” package. The RFE function of the caret package was used to screen the optimal feature subset. The root mean square error (RMSE) was applied to evaluate the performance of different feature subsets. The optimal feature subset that exhibited the lowest RMSE was selected. The overlapped genes selected by LASSO, SVM-RFE, and MCC algorithms were considered candidate aging-related hub genes. These genes were used for further diagnostic analysis of AF, and receiver operating characteristic (ROC) curves were plotted via “pROC” package.

### Analysis of immune cell infiltration

CIBERSORT, a deconvolution algorithm, was used to quantify immune cell infiltration (22 various cell types) in AF gene expression profiles [28]. Pearson’s correlation analysis was applied to calculate the correlation between infiltrating immune cells. The “corrplot” package was used to perform the correlation analysis and visualization of various infiltrating immune cells. The “ggplot2” package was applied to visualize and draw the boxplot of the differences in infiltrating immune cells between AF and SR samples.

### Correlation analysis between aging-related hub genes and infiltrating immune cells

Person correlation analysis was used to investigate in-depth scrutiny of the relationships between aging-related hub genes and infiltrating immune cells. The visualization of correlation analysis was carried out by the “ggplot2” package.

### The establishment of aged AF canine models

The animal experiments were performed with approval and according to the Institutional Animal Care and Use Committee of the Second Xiangya Hospital of Central South University (the ethics number was 2020635). The animal experiments were also performed in accordance with the ARRIVE guidelines. Eight canines (age more than 8 years, 10 kg) were randomly divided into the sham-operated group (Sham, n = 3) and rapid atrial pacing (RAP, n = 5). Aged AF canine models were established by using long-term RAP [29]. Briefly, a programmable pacemaker (AOO, Harbin University of Science and Technology) was implanted and applied for continuous atria pacing at 400 bpm for 8 weeks to induce AF. Electrocardiography was applied to confirm the success of this procedure. Sham-operated group aged canines were implanted with the same pacemaker without activation.

### Real-time PCR assay

Left atrial samples were obtained from sham and RAP-induced AF canine models. Total RNA was extracted from left atrial samples using TransZol Up (ET111-01, Transgenbiotech). cDNA was synthesized using RevertAid First Strand cDNA Synthesis Kit (K1622, Thermo Fisher Scientific). Quantitative real-time PCR (qPCR) reactions were conducted using SYBR Green (A25742, Thermo Fisher Scientific). The 2^-△△Ct^ method was applied to analyze relative mRNA expression normalized to β-actin mRNA levels. The primer sequences are listed in **S3 Table in S1 File**.

### Statistical analysis

All statistical analyses were performed in R (version 4.1.2). The significance of the differential aging-related genes expression was analyzed by the Wilcoxon rank sum test. ROC curves were used to assess the diagnostic efficacy of aging-related hub genes. Pearson correlation analysis was applied to analyze the association between aging-related hub genes and infiltrating immune cells. The student t-test was performed to compare gene expression levels between the two groups.

## Results

### Screening aging-related DEGs in AF

**Fig 2:**
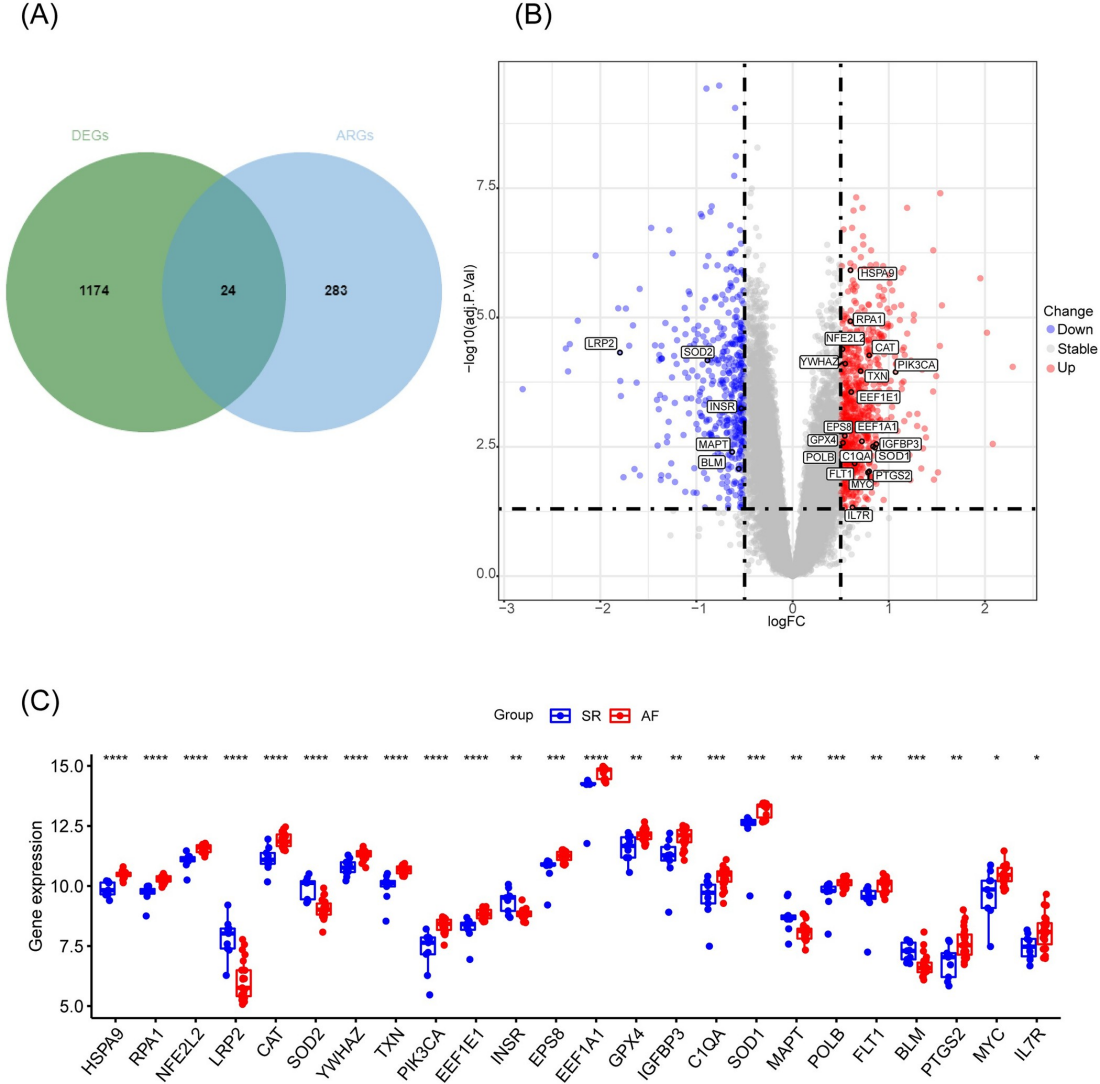
Differential expressed aging-related genes in AF and SR samples. (A) Venn plot of overlapped genes between DEGs from metadata cohort and 307 aging-related genes from Human Aging Genomic Resource. The intersected 24 genes were considered aging-related DEGs. (B)Volcano plot of 24 differential expressed aging-related DEGs. (C)Box plot of 24 differentially expressed aging-related genes in AF and SR samples. *p<0.05, **p<0.01, ***p<0.001, ****p<0.0001.

Differential expression analysis was conducted between 23 AF samples and 9 SR samples in the metadata cohort, comprising GSE79768 and GSE41177 datasets, utilizing the ’limma’ R package. The overlapped genes between DEGs and aging-related genes were considered aging-related DEGs (**Fig 2A and 2B**). A total of 24 aging-related genes were identified (**S4 Table in S1 File)**. Among these 24 aging-related DEGs, 19 genes exhibited significant upregulation, while 5 genes showed significant downregulation (**Fig 2B**). The box plot illustrated their expression patterns in the AF and SR samples (**Fig 2C**). *SOD1*, *IGFBP3*, and *PIK3CA* were the top three upregulated genes, while *LRP2*, *SOD2*, and *MAPT* were the top three downregulated genes. The correlation analysis demonstrated significant intercorrelations among the 24 aging-related DEGs in the metadata cohort (**S1 Fig in S1 File**). Most of these genes exhibited positive correlations; for instance, *HSPA9* displayed positive correlations with *SOD1*, *GPX4*, *IGFBP3*, and *CAT*, but a negative correlation with *SOD2*.

### Functional enrichment analysis of aging-related DEGs

**Fig 3:**
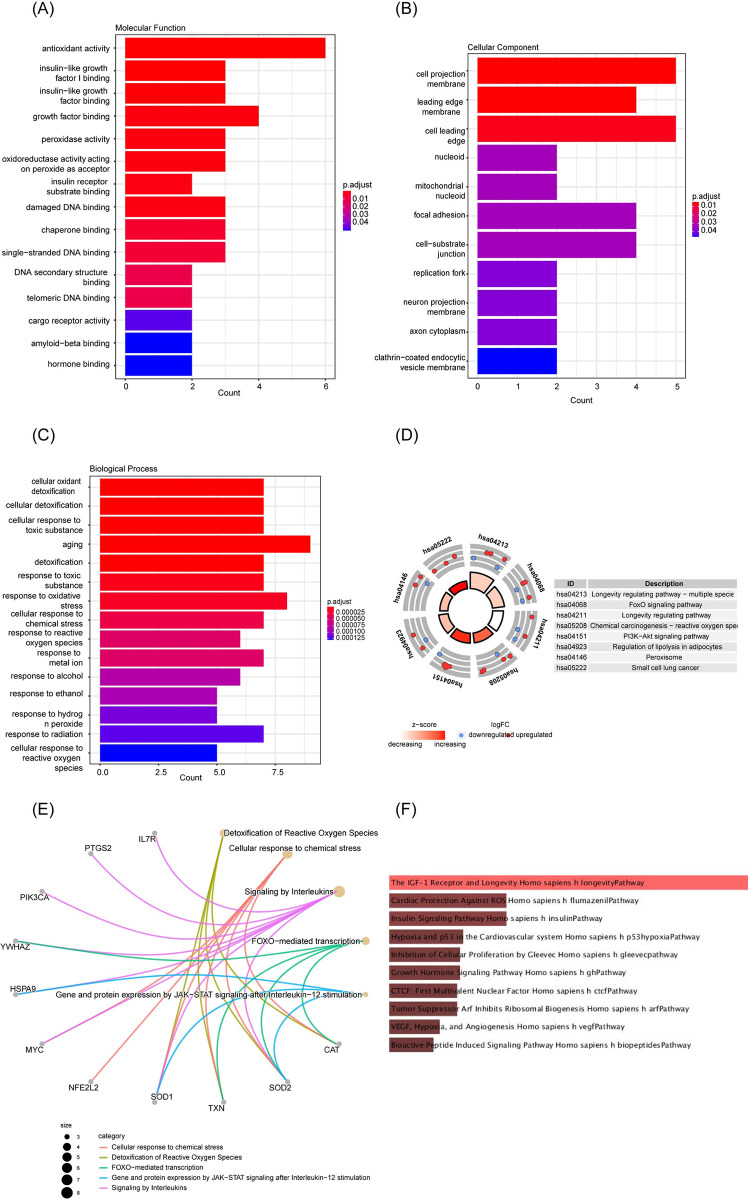
Functional enrichment analysis of 24 differentially expressed aging-related genes. (A) Molecular function. (B) Cellular component. (C) Biological process. (D) Kyoto Encyclopedia ofGenes and Genomes analysis. (E) Reactome pathway analysis. (F) BioCarta pathway analysis.

GO and KEGG pathway analysis were performed to identify the potential biological function of aging-related DEGs. GO enrichment analysis included biological process, molecular function, and cellular component. In the biological process, most of the significant enrichment terms were associated with the regulation of oxidant reactions, such as cellular oxidant detoxication, response to oxidative reaction, and response to oxidative stress. In terms of cellular components, the significant enrichment terms included the cell projection membrane, leading-edge membrane, and cell leading edge. As for molecular functions, the enriched terms included antioxidant activity, binding to insulin-like growth factor I, and peroxidase activity (**Fig 3A–3C**). Furthermore, the results of KEGG enrichment analysis indicated that aging-related DEGs were implicated in several critical pathways, namely, the longevity-regulating pathway, the FoxO signaling pathway, the PI3K-Akt signaling pathway, and peroxisome-related processes (**Fig 3D**). Reactome pathway analysis suggested that these genes were primarily involved in detoxification of reactive oxygen species, cellular response to chemical stress, signaling by interleukins, and FoXO-mediated transcription (**Fig 3E**). Additionally, Additionally, BioCarta pathway analysis highlighted enriched terms related to the IGF-1 receptor and longevity, cardiac protection against ROS, and the insulin signaling pathway (**Fig 3F**).

### Drug prediction for aging-related DEGs

The aging-related DEGs were inputted into the CMap database to identify potential small molecule compounds. As shown in **S5 Table in S1 File**, 7 small molecule compounds with negative Raw_cs and top fdr_q_nlog10 values were identified, including memantine, oligomycin-c, azathioprine, megestrol, carbamazepine, BRD-K99615199, and doxapram, which may be developed as potential drugs for the treatment of AF.

### Protein-protein interaction network of aging-related DEGs

**Fig 4:**
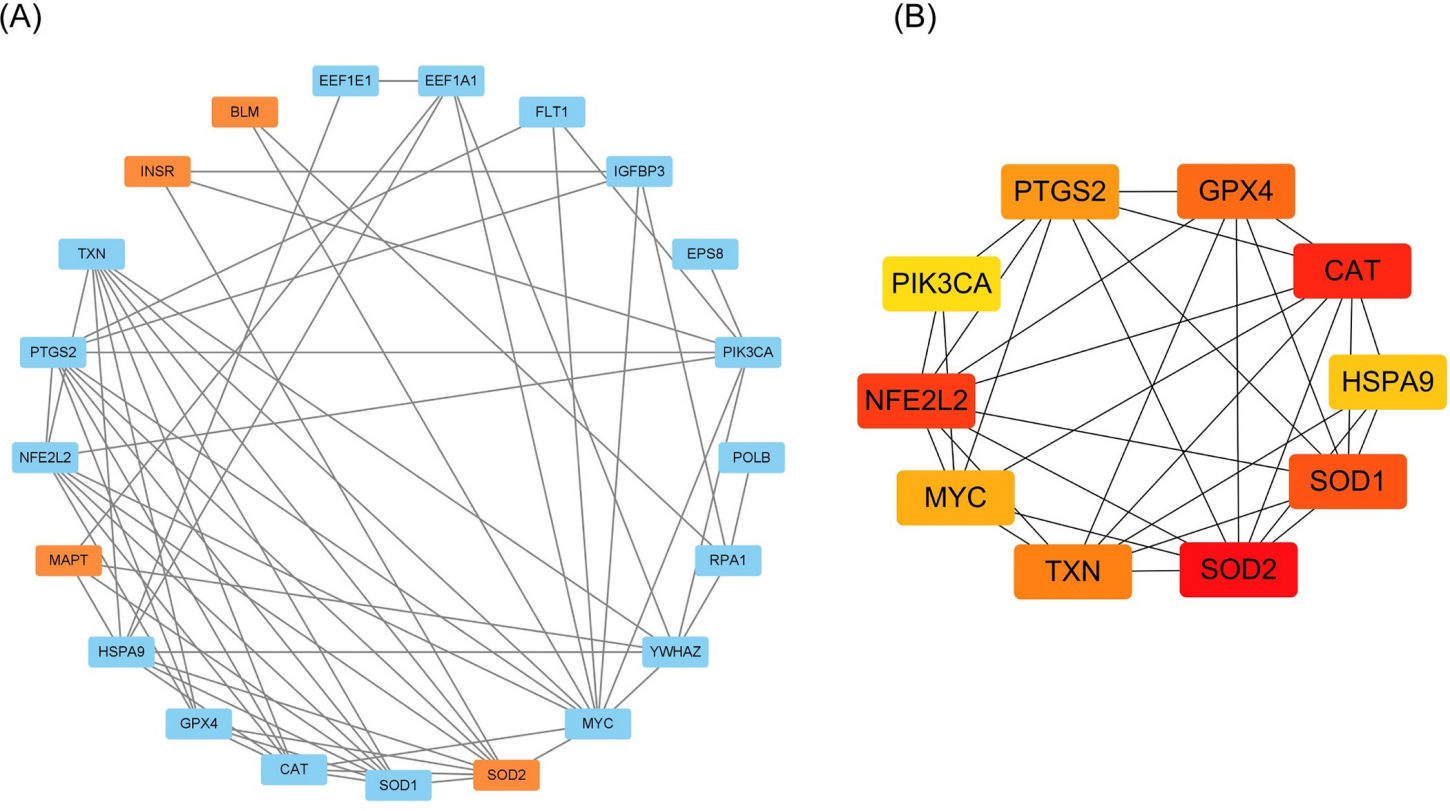
Protein-protein interactions (PPI) analysis of 24 differentially expressed aging-related genes. (A) PPI network of 24 aging-related DEGs. The blue nodes represent the significantly up-regulated genes and the orange nodes represent the significantly down-regulated genes. The edges represent protein-protein interactions. (B) Top 10 genes identified by MCC algorithm via cytohubba. The color of the nodes represented rank. The redder the color, the higher the rank, the oranger the color, the lower the rank.

The PPI network comprises nodes, edges, and their interconnections, with hub genes identified as the most densely interconnected nodes. PPI analysis showed protein functional association among the aging-related DEGs (**Fig 4A**). The top 10 hub genes (*SOD2*, *SOD1*, *HSPA9*, *CAT*, *GPX4*, *PTGS2*, *PIK3A*, *NFE2L2*, *MYC*, and *TXN*) were identified through the MCC algorithm (**Fig 4B**).

### Feature selection of aging-related DEGs

**Fig 5:**
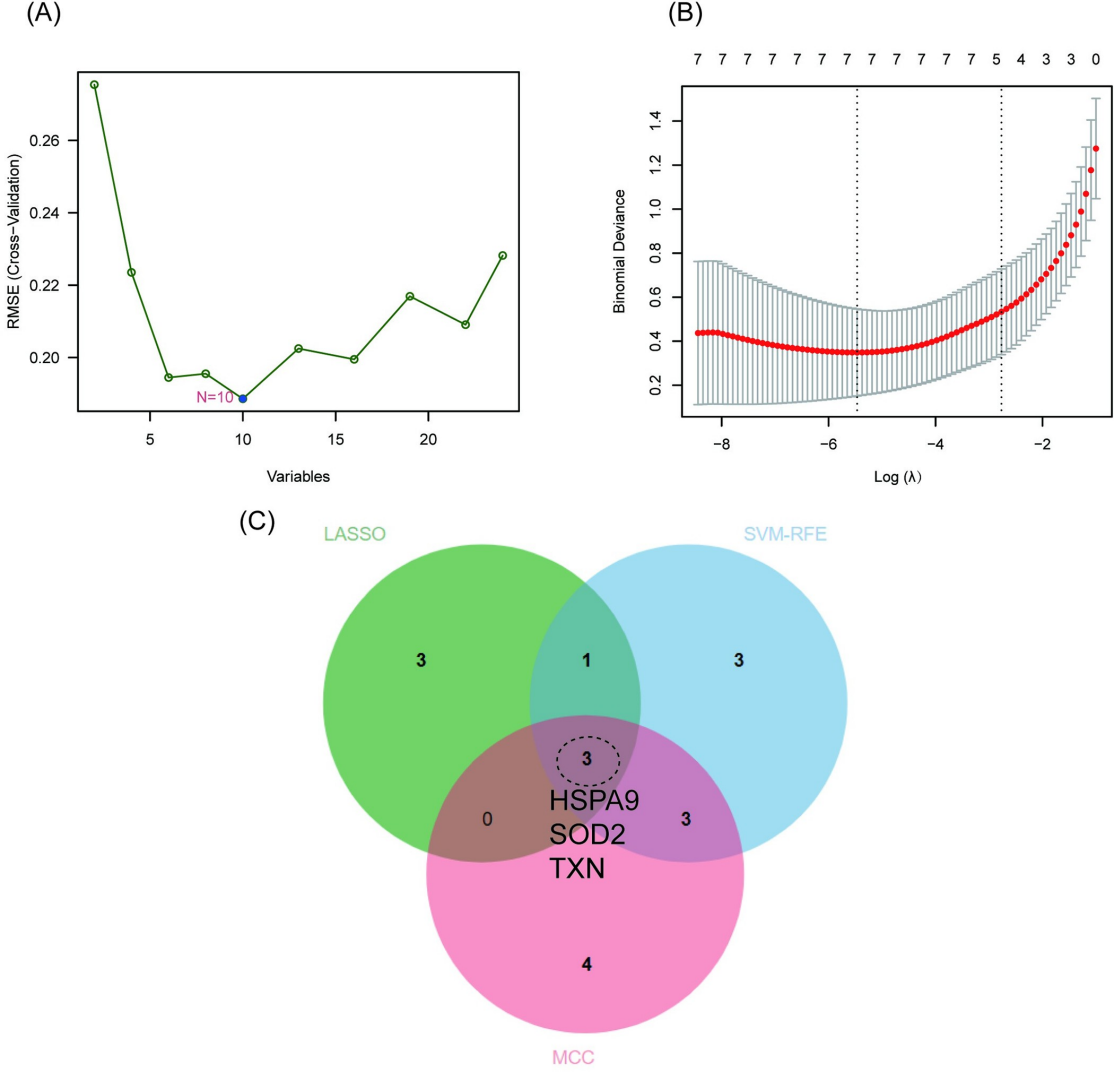
Feature selection of aging-related DEGs. (A) Result of support vector machine-recursive feature elimination (SVM-RFE) algorithm. (B) Result of least absolute shrinkage and selection operator (LASSO) logistic Regression Algorithm. (C) Venn plot of genes selected by LASSO, SVM-RFE and MCC algorithms. HSPA9, SOD2, and TXN were eventually selected.

Aging-related DEGs were further screened by two machine learning algorithms. The SVM-RFE algorithm identified a subset of 10 features in the aging-related DEGs (**Fig 5A**). The LASSO logistic regression algorithm identified 7 genes out of the 24 aging-related DEGs (**Fig 5B**). Subsequently, the intersected genes selected by LASSO, SVM-RFE, and MCC algorithms were considered candidate aging-related hub genes (**Fig 5C**). Among these, *HSPA9*, *SOD2*, and *TXN* were identified.

### Validation of candidate aging-related hub genes

**Fig 6:**
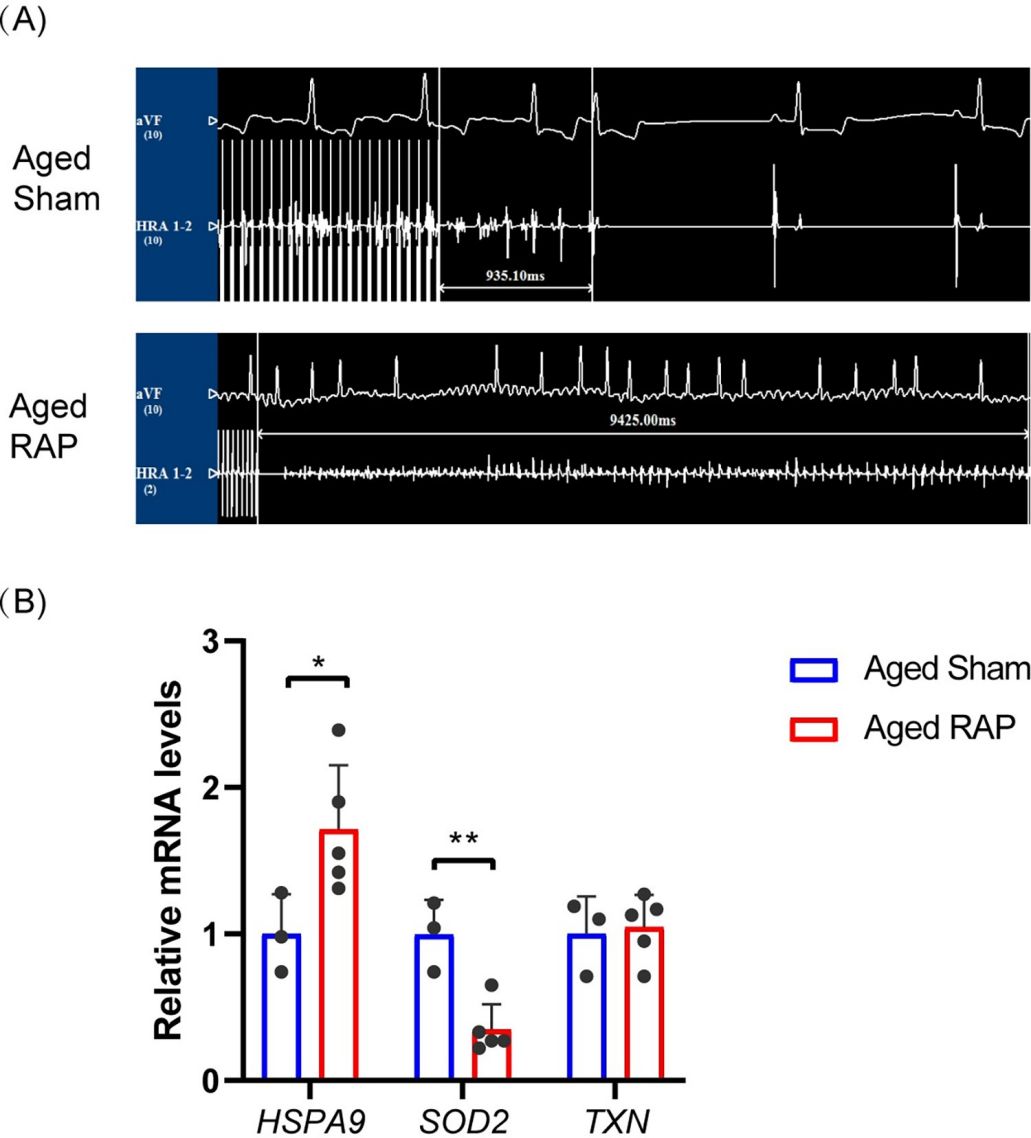
Validation of candidate aging-related hub genes in aged RAP-induced AF canine models. (A) The AF duration in aged sham-operated and RAP canines. (B) The relative mRNA levels of HSPA9, SOD2 and TXN in aged sham-operated and RAP canines. *p<0.05, **p<0.01.

**Fig 7:**
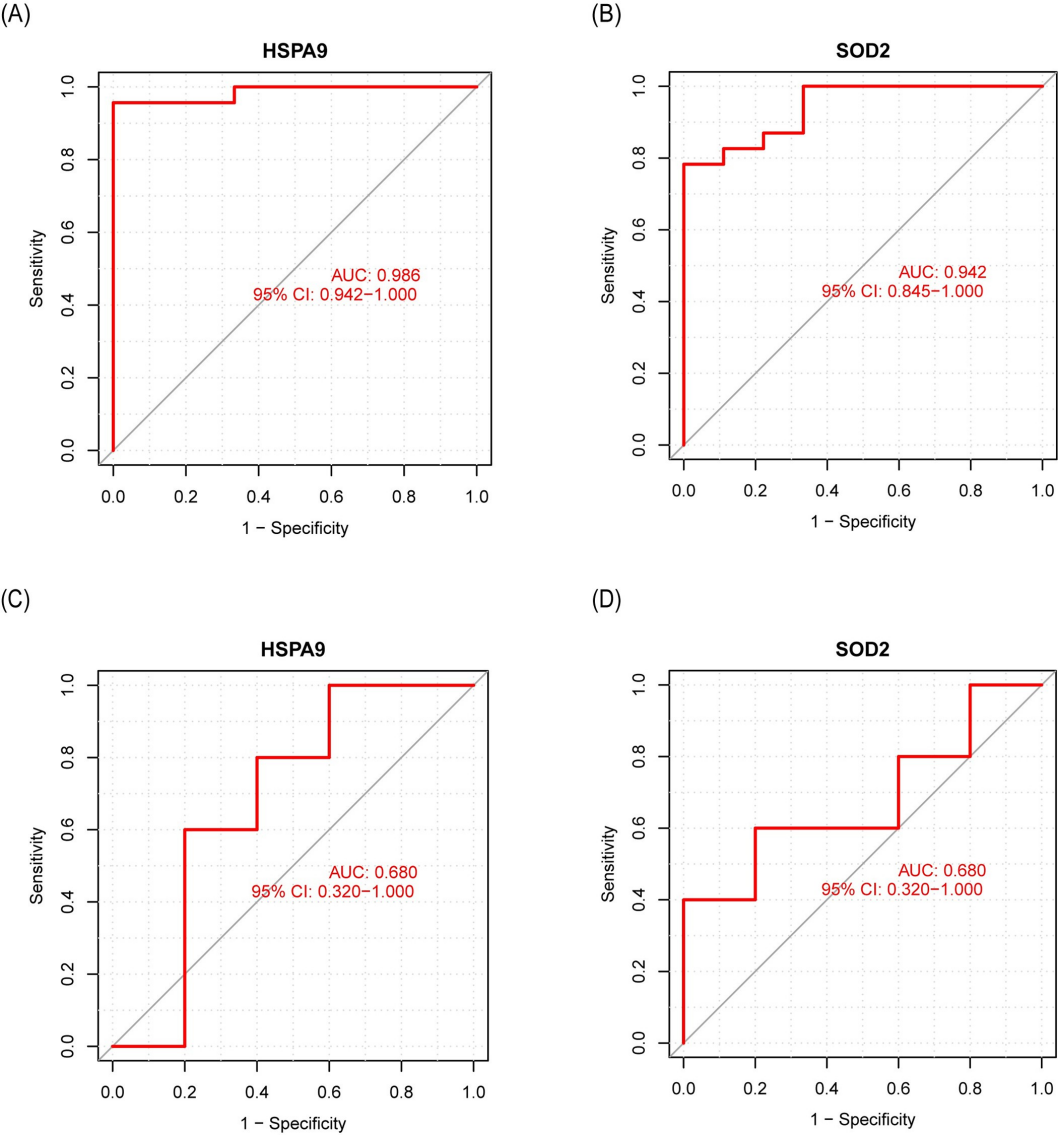
ROC curves of HSPA9 and SOD2 in AF and SR samples. (A, B) ROC curves of HSPA9 and SOD2 in the metadata cohort. (C, D) ROC curves of HSPA9 and SOD2 in the GSE14975 dataset.

The expressions of candidate aging-related hub genes were further explored by qRT-PCR using left atrial samples from aged sham and RAP-induced AF canine models. Electrocardiography results (**Fig 6A**) confirmed the successful establishment of RAP-induced AF canine models. In aged RAP-induced AF canine models, the expression level of *HSPA9* (*p* = 0.047) significantly increased, and the expression level of *SOD2* (*p* = 0.004) significantly decreased, consistent with our bioinformatic analysis (**Fig 6B**). However, the *TXN* expression level was not significantly different between aged sham and RAP-induced AF canine samples. These findings suggested that *HSPA9* and *SOD2* play more prominent roles in age-related AF. Therefore, *TXN* was excluded. Consequently, *HSPA9* and *SOD2* were considered aging-related hub genes. To assess their diagnostic efficacy, ROC curves for *HSPA9* and *SOD2* were constructed using metadata, GSE14975, GSE128188, and GSE115574 datasets, respectively. In the metadata cohort, the area under the curves (AUC) for *HSPA9* and *SOD2* were 0.986 and 0.961, respectively, indicating their high diagnostic value for AF (**Fig 7A and 7B**). The diagnostic efficacy of *HSPA9* and *SOD2* was further validated using the GSE14975, GSE128188, and GSE115574 datasets (**Fig 7C and 7D and S2 Fig in S1 File)**. These findings collectively suggested that *HSPA9* and *SOD2* hold promise as potential diagnostic biomarkers for AF.

### Immune landscape analysis

The CIBERSORT algorithm was applied to calculate the proportion of 22 types of immune cell infiltration in AF and SR samples. CD4^+^ naïve T cells, resting NK cells, macrophages M0 were excluded from analysis as they were not inferred to be expressed in the samples. The results revealed a significant increase in the infiltration levels of resting mast cells, gamma delta T cells, neutrophils, and naïve B cells in AF compared to SR. Conversely, SR showed a higher proportion of infiltration of Macrophages M2, CD8^+^ T cells, and activated NK cells (**S3A Fig in S1 File**). Furthermore, we assessed the correlations among infiltrating immune cells (**S3B Fig in S1 File**). Naïve B cells, plasma cells, and gamma delta T cells showed negative correlations with Macrophages M2. Activated NK cells displayed a positive correlation with CD8^+^ T cells. Furthermore, gamma delta T cells exhibited a positive correlation with naïve B cells but a negative correlation with CD8^+^ T cells.

### Correlation analysis between aging-related hub genes and infiltrating immune cells

**Fig 8:**
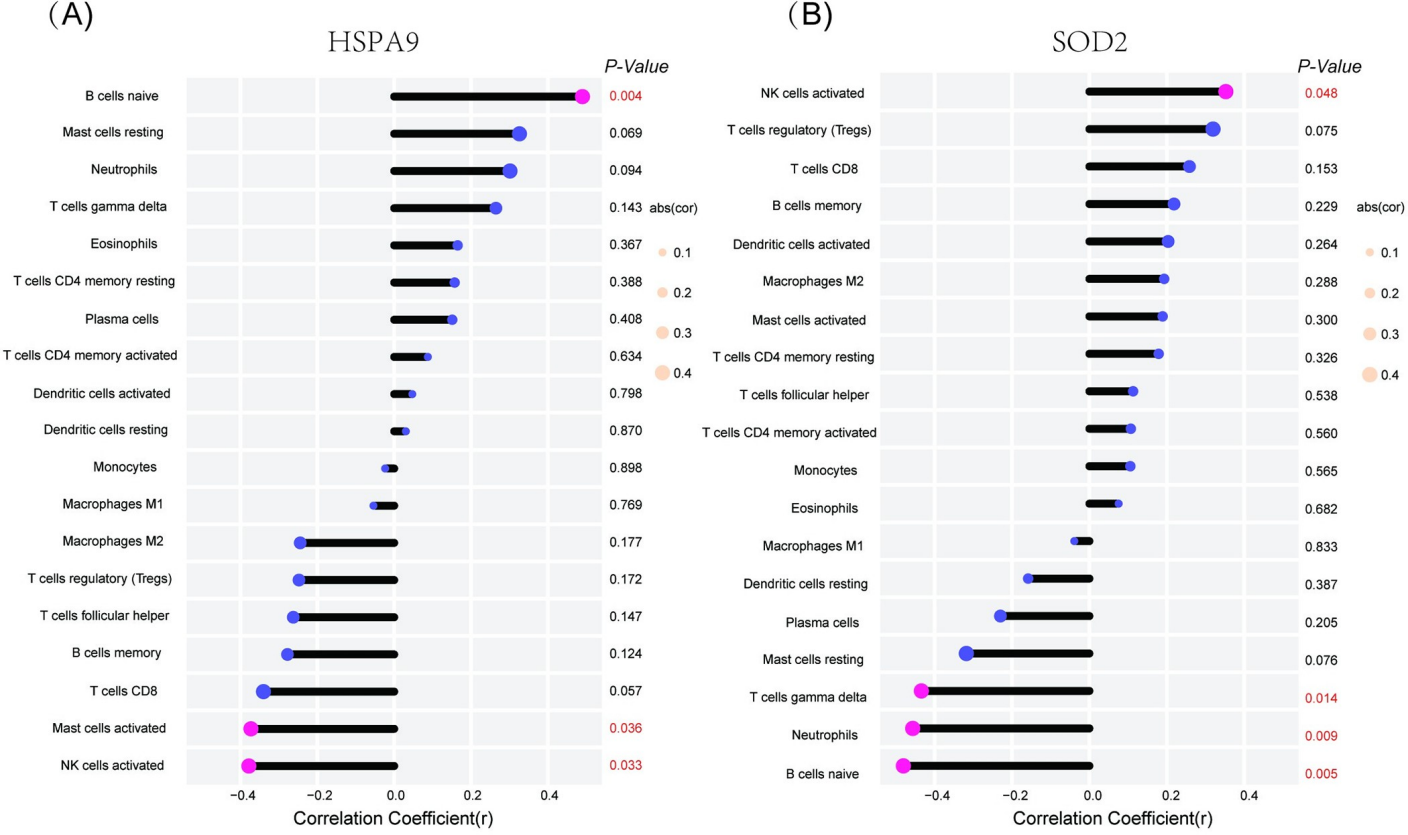
Correlation of HSPA9 and SOD2 with infiltrating immune cell. (A) HSPA9. (B) SOD2. The size of dot represented the strength of the correlation. p<0.05 was considered statistically significant.

Based on the results of correlation analysis, *HSPA9* exhibited a significant positive correlation with naïve B cells (r = 0.49, *p* = 0.004) and a significant negative correlation with activated mast cells (r = -0.37, *p* = 0.036) and activated NK cells (r = -0.38, *p* = 0.033) (**Fig 8A**). Similarly, *SOD2* demonstrated a significant positive correlation with activated NK cells (r = 0.35, *p* = 0.048) and significant negative correlations with gamma delta T cells (r = -0.43, *p* = 0.014), neutrophils (r = -0.46, *p* = 0.009), and naïve B cells (r = -0.48, *p* = 0.005) (**Fig 8B**).

## Discussion

Aging is a well-established risk factor for AF [3], and understanding the underlying mechanism of aging-related AF is crucial for developing new therapeutic strategies. In this study, 24 aging- and AF- related genes were identified. After screening by LASSO, SVM-RFE, and MCC algorithms, *HSPA9*, *SOD2* and *TXN* were selected. Furthermore, aged RAP-induced AF canine models were constructed to verify the expression of these genes. The expression of *HSPA9* and *SOD2* were consistent in aged RAP-induced AF canine models. Consequently, *HSPA9* and *SOD2* were considered aging-related hub genes. ROC curves confirmed *HSPA9* and *SOD2* exhibit remarkable diagnostic capacity for AF.

The GO enrichment analysis highlighted that these genes were principally involved in oxidative stress. Previous studies have shown that oxidative stress could trigger senescence [30]. Recent studies have demonstrated that various proinflammatory processes linked to oxidative stress could promote atrial structure remodeling [31]. For example, mitochondria-derived ROS could cause AF in three transgenic mouse models harboring ryanodine receptor (RyR2) mutations. Moreover, senescent cells secrete inflammation cytokines, growth factors, immune modulators, and matrix metalloproteinases, rendering the senescence-associated secretory phenotype (SASP) [32]. Atrial fibrosis, a prominent feature of atrial structural remodeling, plays a critical role in AF development and maintenance [33]. Previous studies have shown that SASP participates in fibrosis initiation by promoting inflammation [34]. The KEGG enrichment analysis demonstrated that these genes were mainly involved in the FoxO signaling pathway, longevity regulating pathway, PI3K-Akt signaling pathway, and peroxisome. They were associated with AF pathophysiology, such as aging, inflammation, and oxidative stress [5, 31]. Nevertheless, further investigations are warranted to elucidate the precise biological functions of these aging-related genes associated with AF.

*SOD1*, *IGFBP3*, *PLk3CA*, *LRP2*, *SOD2*, and *MAPT* were identified as top dysregulated genes, implying a potentially significant role for these genes in AF pathology. These genes are primarily involved in regulation of oxidative stress and fibrosis, both of which are associated with the development of AF [35, 36]. Following thorough screening with multiple algorithms, *HSPA9* and *SOD2* were conclusively identified as aging-related hub genes. Heat shock protein family A member 9 (*HSPA9*), also known as mortalin, is a stress chaperone that regulates a variety of cellular functions, including stress response, chromosome replication, proliferation, and apoptosis [37]. *HSPA9* directly regulates calcium shuttle from the endoplasmic reticulum (ER) to the mitochondria and maintains calcium homeostasis, preventing excess ROS generation and ER dysfunction [38]. In human bronchial epithelial cells, exposure to high mobility group box 1 (HMGB1) could induce the expression of *HSPA9*, reinforcing ER-mitochondria Ca2^+^ transfer and ROS production. However, the above changes were mitigated via the knockdown of *HSPA9* with *HSPA9* siRNA [39]. Furthermore, inhibition of *HSPA9* in HL-1 cells and deletion of *HSPA9* in cardiomyocytes of diabetic mouse models mitigates mitochondrial oxidative stress and calcium overload [40]. Inactivation of *HSPA9* in *Myh6*-*Cre*^*ERT2*^; *Hspa9*^fl/fl^ mice also alleviated atrial remodeling and AF progression [40]. A previous study demonstrated that the expression of *HSPA9* increased more than twofold in myocardial samples from chronic AF patients compared with SR patients [41]. Consistently, our study observed an increased *HSPA9* expression in aged RAP-induced AF canine models.

Superoxide dismutase 2 (SOD2) is a vital mitochondrion antioxidant enzyme. SOD2 could protect cells against oxidative damage by promoting the transformation of superoxide radicals into oxygen and hydrogen peroxide [42]. Adipokine asprosin could protect mesenchymal stromal cells from oxidative stress-induced apoptosis through the ERK1/2-SOD2 pathway [43]. *SOD2* inactivation increased mitochondrial O_2_^-^• and declined endothelial nitric oxide and was related to hypertension [44]. Moreover, pioglitazone partially inhibits aging-related arrhythmogenic atrial remodeling by promoting the expression of *SOD2* [45]. Our study showed that *SOD2* was downregulated in AF patients and aged RAP-induced AF canine models, which suggested that there may be enhanced oxidative stress in AF. Moreover, the results of the PPI analysis and correlation analysis indicated a close interrelationship between *HSPA9* and *SOD2*, suggesting their potential collaborative role in the onset and progression of AF. Furthermore, *HSPA9* and *SOD2* showed promise for improving AF diagnosis, as evidenced by their higher AUC values in the metadata and other datasets. These findings suggested that *HSPA9* and *SOD2* hold potential as therapeutic targets and novel approaches for detecting AF.

Mounting evidence suggests that immune cell dysfunction is involved in AF pathogenesis [13]. Herein, immune cell infiltration was analyzed across AF left atrial tissues. Our findings revealed a decrease in the infiltration of macrophages M2 and activated NK cells, along with an increase in the infiltration of neutrophils and gamma delta T cells, suggesting potential involvement of these immune cells in AF pathogenesis. Neutrophils, being a primary source of ROS and myeloperoxidase (MPO), may contribute to a pro-fibrotic role in the development of AF [13]. Macrophages may participate in AF development by secreting cytokines like TNF-α and IL-1β [13]. *HSPA9* and *SOD2* were related to diverse immune cell subpopulations across AF atrial tissues, indicating that these identified aging-related genes might regulate the inflammatory response during AF progression.

CMap analysis was conducted to predict small molecule drugs with potential efficacy in addressing aging-related AF. Notable candidates identified include memantine and doxapram. Memantine, a well-established ionotropic glutamate receptors (iGluRs) antagonist primarily employed in the treatment of Alzheimer’s disease [46], has garnered recent attention due to the high expression of iGluRs in atrial cardiomyocytes [47]. Inhibition of iGluRs has demonstrated the ability to reduce AF incidence and alleviate AF progression in AF models using isolated rat hearts [47]. Furthermore, numerous studies have provided evidence of memantine’s capacity to ameliorate oxidative damage in various diseases [48–50]. In human umbilical vein endothelial cells, memantine mitigated inflammation, oxidative stress, and apoptosis induced by oxidized low-density lipoprotein through the activation of the BDNF/TrkB signaling pathway [50]. Based on these findings, we hypothesize that memantine may upregulate *SOD2* and downregulate *HSPA9*, thus mitigating oxidative stress and potentially inhibiting the development of AF. Doxapram, known as a potassium channel antagonist, exhibits significant inhibitory effects on TWIK-related acid-sensitive potassium channel 1 (TASK-1), resulting in the cardioversion of AF in porcine models [51]. These drugs may delay the occurrence and development of AF.

Limitations of our study should be acknowledged. Firstly, we employed qRT-PCR to validate HSPA9 and SOD2 expression in aged RAP-induced AF canine models, despite inherent species differences between humans and dogs. In contrast to humans, dogs have a faster resting heart rate and exhibit a distinct atrial action potential [52]. Although dogs could naturally develop spontaneous AF, it’s worth noting that this phenomenon might be more apparent in dogs due to their relatively higher levels of veterinary care compared to many other species [52]. These distinctions underscore the need for caution when generalizing results obtained from dogs to humans. Our choice of aged canines for gene expression validation stems from the challenge of obtaining human left atrial samples. Nonetheless, canines have seen extensive use in AF research [52]. In an ATP canine model, sympathovagal denervation successfully abolished paroxysmal AF episodes, highlighting the role of sympathovagal input in AF initiation [53]. Secondly, our findings primarily revolve around gene expression analysis. Although gene expression doesn’t directly mirror protein expression, additional investigations are imperative to elucidate the biological functions and underlying mechanisms of these genes. Lastly, our study’s dataset comprised a relatively modest sample size. Future research with larger cohorts is warranted. We remain committed to monitoring these identified genes to advance our comprehension of the pathogenesis and therapeutic strategies for aging-related AF.

## Conclusion

*HSPA9* and *SOD2* were identified via comprehensive bioinformatics analysis and validation of aged RAP-induced AF canine models in this study. *HSPA9* and *SOD2* were not only related to aging but may also be involved in the regulation of the atrial immune response of AF patients. Our findings expand knowledge regarding the underlying mechanisms of aging-related AF.

## Supporting information

S1 File (DOCX)

S2 File (DOCX)
