# Supplementary material for: Identification and validation of aging-related genes in atrial fibrillation
Source: PLoS One. 2023 Nov 13;18(11):e0294282. doi: 10.1371/journal.pone.0294282 (PMC10642816; doi:10.1371/journal.pone.0294282)
Supplement: S1 File — (DOCX) [file pone.0294282.s001.docx]

Supplementary file

Identification and Validation of Aging-Related Genes in Atrial Fibrillation

**Supplementary Table S1. Basic information of the patients in GSE79768 and GSE41177**

|  | **GSE79768** | | **GSE41177** | |
| --- | --- | --- | --- | --- |
|  | **AF (7)** | **SR (6)** | **AF (16)** | **SR (3)** |
| **Age** | 48 ± 11 | 64 ± 15 | 54 ± 13 | 53 ± 10 |
| **Sex, M : F** | 3:4 | 2:4 | 8:8 | 2:1 |
| **Ejection fraction (%)** | 60 ± 11 | 52 ± 21 | 60 ± 9 | 55 ± 7 |
| **Left arterial diameter (mm)** | 59 ± 10 | 48 ± 10 | 54 ± 6 | 48 ± 5 |
| **Hypertension** | 1 | 3 | 4 | 2 |
| **Diabetes mellitus** | 0 | 2 | 3 | 2 |

AF = atrial fibrillation, SR=sinus rhythm, M: F = male : female; data are presented as mean ± SEM

**Supplementary Table S3. The sequences of primers used for quantitative real-time PCR**

| **Genes** | **Sequences** |
| --- | --- |
| β-actin | Forward: tgcgtgacatcaaggagaag |
|  | Reverse: aggaaggaaggctggaagag |
| HSPA9 | Forward: ATTGATGCCAATGGGATTGT |
|  | Reverse: taactgcttcaacccgttcc |
| SOD2 | Forward: gggttggcttggtttcaata |
|  | Reverse: tgcaagccatgtatctctcg |
| TXN | Forward: atgcttttcaggaagccttg |
|  | Reverse: acccaccttttgtcccttct |

**Supplementary Table S4. The 24 differentially expressed aging-related genes**

| **Gene Symbol** | **logFC** | **Changes** | **P.Value** | **adj.P.Val** |
| --- | --- | --- | --- | --- |
| HSPA9 | 0.60197 | Up | 4.16E-09 | 1.22E-06 |
| RPA1 | 0.601678 | Up | 1.39E-07 | 1.18E-05 |
| NFE2L2 | 0.518397 | Up | 9.74E-07 | 4.08E-05 |
| LRP2 | -1.79569 | Down | 1.20E-06 | 4.76E-05 |
| CAT | 0.796608 | Up | 1.48E-06 | 5.39E-05 |
| SOD2 | -0.88547 | Down | 2.07E-06 | 6.72E-05 |
| YWHAZ | 0.547129 | Up | 2.56E-06 | 7.83E-05 |
| TXN | 0.707138 | Up | 4.10E-06 | 0.000109 |
| PIK3CA | 1.071205 | Up | 4.38E-06 | 0.000113 |
| EEF1E1 | 0.609626 | Up | 1.55E-05 | 0.000275 |
| INSR | -0.53447 | Down | 4.28E-05 | 0.000571 |
| EPS8 | 0.542307 | Up | 0.00024 | 0.001936 |
| EEF1A1 | 0.720484 | Up | 0.000334 | 0.002473 |
| GPX4 | 0.523832 | Up | 0.000369 | 0.002649 |
| IGFBP3 | 0.869829 | Up | 0.0004 | 0.002801 |
| C1QA | 0.836651 | Up | 0.00046 | 0.003106 |
| SOD1 | 0.85679 | Up | 0.000497 | 0.003283 |
| MAPT | -0.62894 | Down | 0.000637 | 0.003973 |
| POLB | 0.517165 | Up | 0.00082 | 0.004779 |
| FLT1 | 0.644833 | Up | 0.001269 | 0.006618 |
| BLM | -0.55971 | Down | 0.001757 | 0.008407 |
| PTGS2 | 0.796975 | Up | 0.002054 | 0.009438 |
| MYC | 0.783005 | Up | 0.002191 | 0.009916 |
| IL7R | 0.622703 | Up | 0.016803 | 0.046852 |

**Supplementary Table S5. Identified 7 small molecular drugs by CMap**

| Rank | CMAP name | MOA | Raw_cs | fdr_q_nlog10 |
| --- | --- | --- | --- | --- |
| 1 | memantine | Glutamate receptor antagonist | -0.80 | 15.65 |
| 2 | oligomycin-c | ATP synthase inhibitor | -0.79 | 15.65 |
| 3 | azathioprine | Dehydrogenase inhibitor | -0.78 | 15.65 |
| 4 | megestrol | Progesterone receptor agonist | -0.78 | 15.65 |
| 5 | carbamazepine | Carboxamide antiepileptic | -0.76 | 15.65 |
| 6 | BRD-K99615199 | Progesterone receptor agonist | -0.75 | 15.65 |
| 7 | doxapram | Potassium channel antagonist | -0.74 | 15.65 |


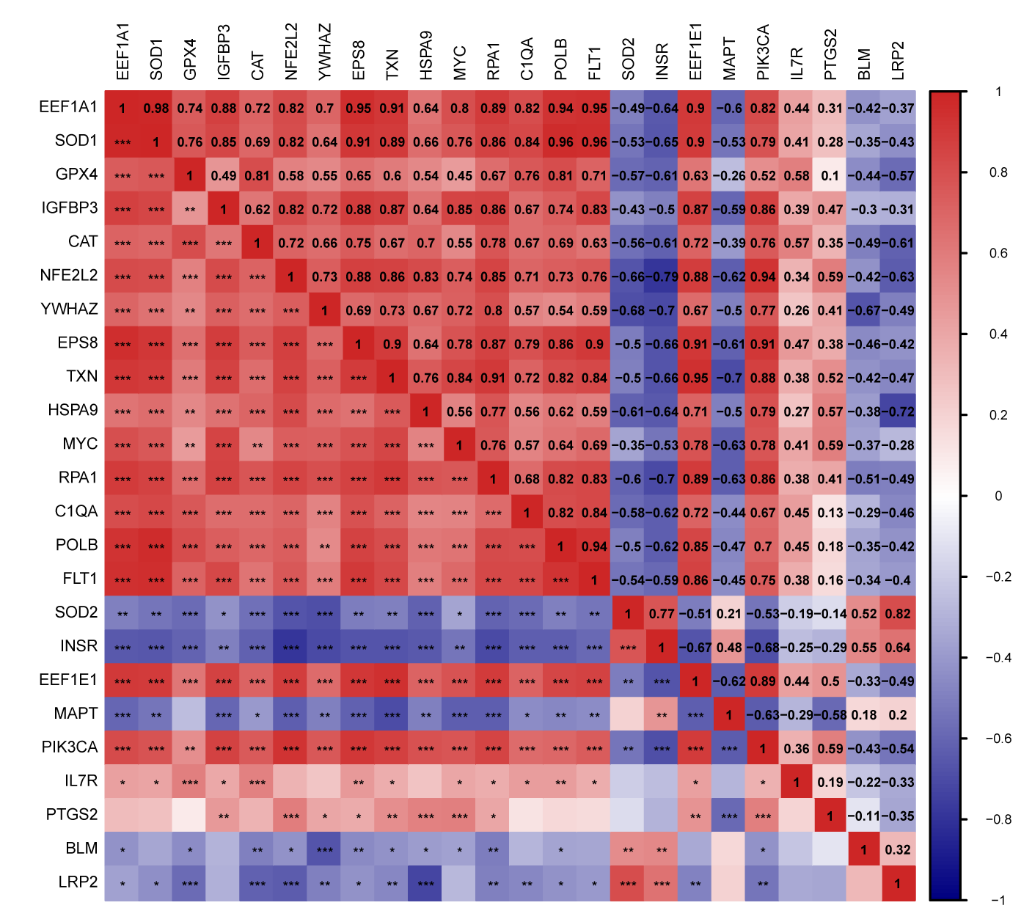


**Supplementary Figure S1. Pearson correlation analysis of 24 differentially expressed aging-related genes.**

Red represented positive correlation, blue represented negative correlation. *p<0.05, **p<0.01, ***p<0.001, ****p<0.0001.


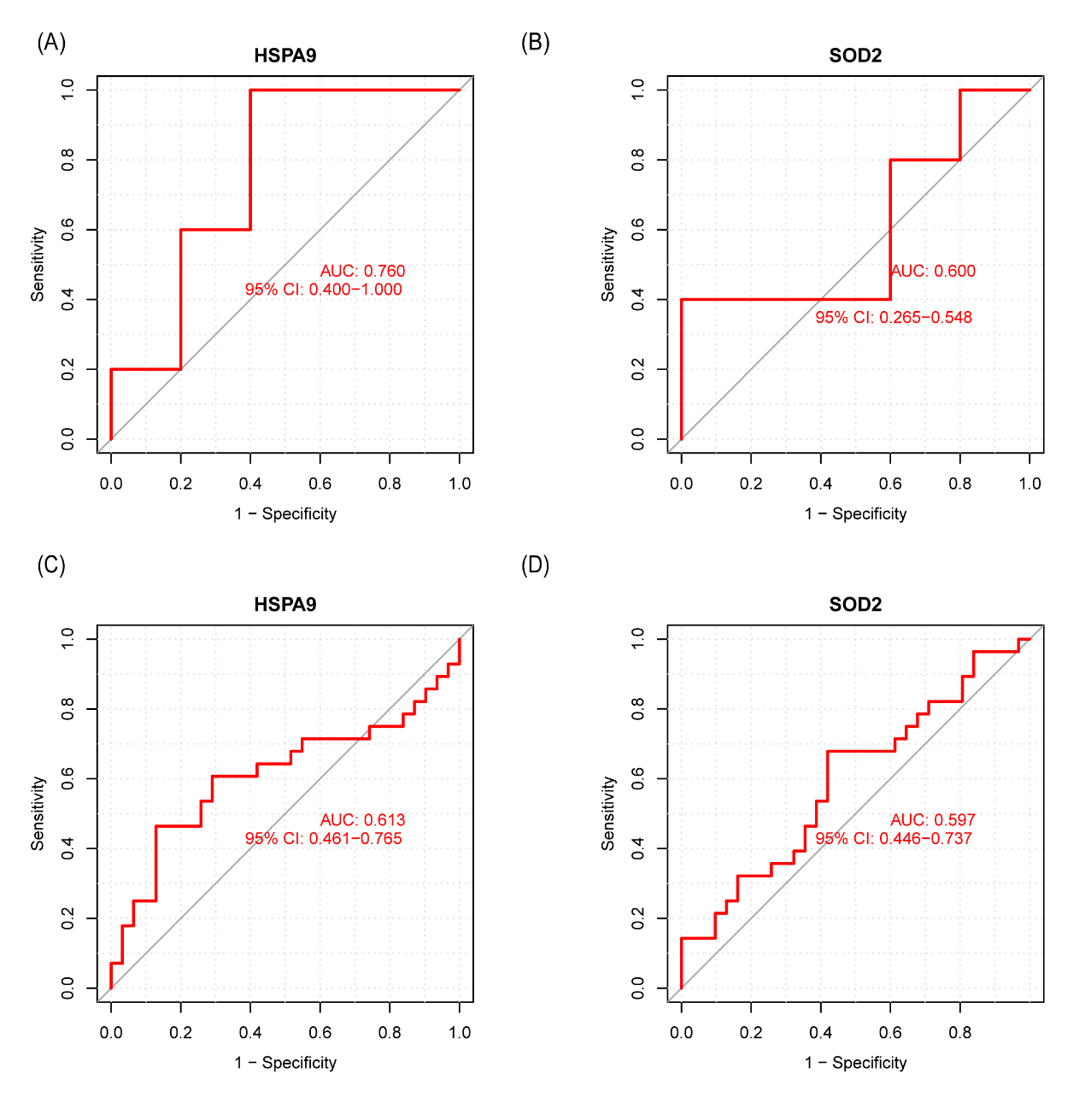


**Supplementary Figure S2.** **ROC curves of *HSPA9* and *SOD2* in AF and SR samples.**

(A, B) ROC curves of *HSPA9* and *SOD2* in the GSE128188 cohort. (C, D) ROC curves of *HSPA9*, and *SOD2* in the GSE115574 dataset.


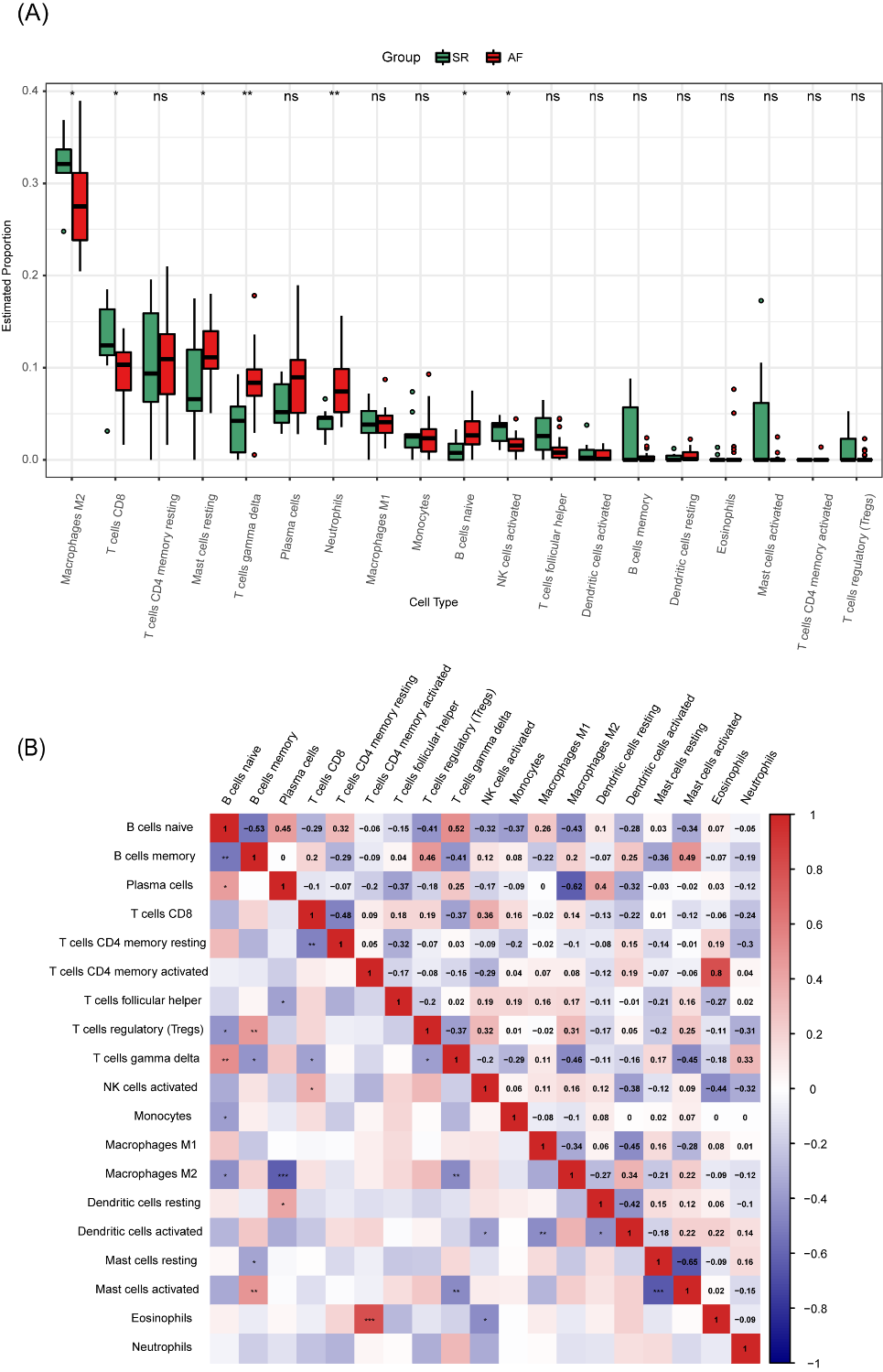


**Supplementary Figure S3. Landscape of immune cell infiltration in AF.**

1. Boxplot of the proportion of immune cell infiltrates. *P < 0.05, **P < 0.01, ns, not

significant. (B) Heatmap plot of correlation among immune cells. Red indicates positive correlation and blue indicates negative correlation.
