# Supplementary material for: Identification and validation of aging-related genes in atrial fibrillation
Source: PLoS One. 2023 Nov 13;18(11):e0294282. doi: 10.1371/journal.pone.0294282 (PMC10642816; doi:10.1371/journal.pone.0294282)
Supplement: S2 File — (DOCX) [file pone.0294282.s002.docx]

| **Supplementary Table S2. There hundred and seven genes related to aging** | | | | |  |
| --- | --- | --- | --- | --- | --- |
| **ID** | **Symbol** | **Name** | **Entrez gene id** | **Uniprot** | |
| 1 | GHR | growth hormone receptor | 2690 | GHR_HUMAN | |
| 2 | GHRH | growth hormone releasing hormone | 2691 | SLIB_HUMAN | |
| 3 | SHC1 | SHC (Src homology 2 domain containing) transforming protein 1 | 6464 | SHC1_HUMAN | |
| 4 | POU1F1 | POU class 1 homeobox 1 | 5449 | PIT1_HUMAN | |
| 5 | PROP1 | PROP paired-like homeobox 1 | 5626 | PROP1_HUMAN | |
| 6 | TP53 | tumor protein p53 | 7157 | P53_HUMAN | |
| 7 | TERC | telomerase RNA component | 7012 | TERC | |
| 8 | TERT | telomerase reverse transcriptase | 7015 | TERT_HUMAN | |
| 9 | ATM | ATM serine/threonine kinase | 472 | ATM_HUMAN | |
| 10 | PLAU | plasminogen activator, urokinase | 5328 | UROK_HUMAN | |
| 11 | ERCC2 | excision repair cross-complementation group 2 | 2068 | ERCC2_HUMAN | |
| 12 | ERCC8 | excision repair cross-complementation group 8 | 1161 | ERCC8_HUMAN | |
| 13 | WRN | Werner syndrome, RecQ helicase-like | 7486 | WRN_HUMAN | |
| 14 | LMNA | lamin A/C | 4000 | LMNA_HUMAN | |
| 15 | IGF1R | insulin-like growth factor 1 receptor | 3480 | IGF1R_HUMAN | |
| 16 | TXN | thioredoxin | 7295 | THIO_HUMAN | |
| 17 | KL | klotho | 9365 | KLOT_HUMAN | |
| 18 | E2F1 | E2F transcription factor 1 | 1869 | E2F1_HUMAN | |
| 19 | PTPN11 | protein tyrosine phosphatase, non-receptor type 11 | 5781 | PTN11_HUMAN | |
| 20 | NFKB2 | nuclear factor of kappa light polypeptide gene enhancer in B-cells 2 (p49/p100) | 4791 | NFKB2_HUMAN | |
| 21 | STAT5B | signal transducer and activator of transcription 5B | 6777 | STA5B_HUMAN | |
| 22 | STAT3 | signal transducer and activator of transcription 3 (acute-phase response factor) | 6774 | STAT3_HUMAN | |
| 23 | STAT5A | signal transducer and activator of transcription 5A | 6776 | STA5A_HUMAN | |
| 24 | NRG1 | neuregulin 1 | 3084 | NRG1_HUMAN | |
| 25 | HDAC3 | histone deacetylase 3 | 8841 | HDAC3_HUMAN | |
| 26 | GH1 | growth hormone 1 | 2688 | SOMA_HUMAN | |
| 27 | IL7R | interleukin 7 receptor | 3575 | IL7RA_HUMAN | |
| 28 | IGF1 | insulin-like growth factor 1 (somatomedin C) | 3479 | IGF1 | |
| 29 | IGF2 | insulin-like growth factor 2 | 3481 | IGF2_HUMAN | |
| 30 | INS | insulin | 3630 | INS_HUMAN | |
| 31 | NGF | nerve growth factor (beta polypeptide) | 4803 | NGF_HUMAN | |
| 32 | IRS1 | insulin receptor substrate 1 | 3667 | IRS1_HUMAN | |
| 33 | PTPN1 | protein tyrosine phosphatase, non-receptor type 1 | 5770 | PTN1_HUMAN | |
| 34 | IRS2 | insulin receptor substrate 2 | 8660 | IRS2_HUMAN | |
| 35 | AKT1 | v-akt murine thymoma viral oncogene homolog 1 | 207 | AKT1_HUMAN | |
| 36 | PIK3CB | phosphatidylinositol-4,5-bisphosphate 3-kinase, catalytic subunit beta | 5291 | PK3CB_HUMAN | |
| 37 | NGFR | nerve growth factor receptor | 4804 | TNR16_HUMAN | |
| 38 | HRAS | Harvey rat sarcoma viral oncogene homolog | 3265 | RASH_HUMAN | |
| 39 | MYC | v-myc avian myelocytomatosis viral oncogene homolog | 4609 | MYC_HUMAN | |
| 40 | EGFR | epidermal growth factor receptor | 1956 | EGFR_HUMAN | |
| 41 | ERBB2 | erb-b2 receptor tyrosine kinase 2 | 2064 | ERBB2_HUMAN | |
| 42 | INSR | insulin receptor | 3643 | INSR_HUMAN | |
| 43 | NCOR1 | nuclear receptor corepressor 1 | 9611 | NCOR1_HUMAN | |
| 44 | NBN | nibrin | 4683 | NBN_HUMAN | |
| 45 | JUND | jun D proto-oncogene | 3727 | JUND_HUMAN | |
| 46 | IL2 | interleukin 2 | 3558 | IL2_HUMAN | |
| 47 | PDGFB | platelet-derived growth factor beta polypeptide | 5155 | PDGFB_HUMAN | |
| 48 | EGF | epidermal growth factor | 1950 | EGF_HUMAN | |
| 49 | IL2RG | interleukin 2 receptor, gamma | 3561 | IL2RG_HUMAN | |
| 50 | FOS | FBJ murine osteosarcoma viral oncogene homolog | 2353 | FOS_HUMAN | |
| 51 | PDGFRB | platelet-derived growth factor receptor, beta polypeptide | 5159 | PGFRB_HUMAN | |
| 52 | EPOR | Erythropoietin receptor | 2057 | EPOR_HUMAN | |
| 53 | SST | somatostatin | 6750 | SMS_HUMAN | |
| 54 | PRKCD | protein kinase C, delta | 5580 | KPCD_HUMAN | |
| 55 | PPARA | peroxisome proliferator-activated receptor alpha | 5465 | PPARA_HUMAN | |
| 56 | RET | ret proto-oncogene | 5979 | RET_HUMAN | |
| 57 | PLCG2 | phospholipase C, gamma 2 (phosphatidylinositol-specific) | 5336 | PLCG2_HUMAN | |
| 58 | PEX5 | peroxisomal biogenesis factor 5 | 5830 | PEX5_HUMAN | |
| 59 | TCF3 | transcription factor 3 | 6929 | TFE2_HUMAN | |
| 60 | PARP1 | poly (ADP-ribose) polymerase 1 | 142 | PARP1_HUMAN | |
| 61 | BRCA1 | breast cancer 1, early onset | 672 | BRCA1_HUMAN | |
| 62 | PIN1 | peptidylprolyl cis/trans isomerase, NIMA-interacting 1 | 5300 | PIN1_HUMAN | |
| 63 | PTEN | phosphatase and tensin homolog | 5728 | PTEN_HUMAN | |
| 64 | CREBBP | CREB binding protein | 1387 | CBP_HUMAN | |
| 65 | HIF1A | hypoxia inducible factor 1, alpha subunit (basic helix-loop-helix transcription factor) | 3091 | HIF1A_HUMAN | |
| 66 | UBB | ubiquitin B | 7314 | UBIQ_HUMAN | |
| 67 | RPA1 | replication protein A1, 70kDa | 6117 | RFA1_HUMAN | |
| 68 | BLM | Bloom syndrome, RecQ helicase-like | 641 | BLM_HUMAN | |
| 69 | BCL2 | B-cell CLL/lymphoma 2 | 596 | BCL2_HUMAN | |
| 70 | S100B | S100 calcium binding protein B | 6285 | S100B_HUMAN | |
| 71 | VCP | valosin containing protein | 7415 | TERA_HUMAN | |
| 72 | POLG | polymerase (DNA directed), gamma | 5428 | DPOG1_HUMAN | |
| 73 | IGFBP3 | insulin-like growth factor binding protein 3 | 3486 | IBP3_HUMAN | |
| 74 | HSP90AA1 | heat shock protein 90kDa alpha (cytosolic), class A member 1 | 3320 | HS90A_HUMAN | |
| 75 | NR3C1 | nuclear receptor subfamily 3, group C, member 1 (glucocorticoid receptor) | 2908 | GCR_HUMAN | |
| 76 | EGR1 | early growth response 1 | 1958 | EGR1_HUMAN | |
| 77 | VEGFA | vascular endothelial growth factor A | 7422 | VEGFA_HUMAN | |
| 78 | ABL1 | ABL proto-oncogene 1, non-receptor tyrosine kinase | 25 | ABL1_HUMAN | |
| 79 | BRCA2 | breast cancer 2, early onset | 675 | BRCA2_HUMAN | |
| 80 | TOP2A | topoisomerase (DNA) II alpha | 7153 | TOP2A_HUMAN | |
| 81 | TOP2B | topoisomerase (DNA) II beta | 7155 | TOP2B_HUMAN | |
| 82 | NFKB1 | nuclear factor of kappa light polypeptide gene enhancer in B-cells 1 | 4790 | NFKB1_HUMAN | |
| 83 | TOP1 | topoisomerase (DNA) I | 7150 | TOP1_HUMAN | |
| 84 | RAD51 | RAD51 recombinase | 5888 | RAD51_HUMAN | |
| 85 | UBE2I | ubiquitin-conjugating enzyme E2I | 7329 | UBC9_HUMAN | |
| 86 | TNF | tumor necrosis factor | 7124 | TNFA_HUMAN | |
| 87 | PDPK1 | 3-phosphoinositide dependent protein kinase 1 | 5170 | PDPK1_HUMAN | |
| 88 | CEBPA | CCAAT/enhancer binding protein (C/EBP), alpha | 1050 | CEBPA_HUMAN | |
| 89 | CEBPB | CCAAT/enhancer binding protein (C/EBP), beta | 1051 | CEBPB_HUMAN | |
| 90 | MXI1 | MAX interactor 1, dimerization protein | 4601 | MXI1_HUMAN | |
| 91 | TGFB1 | transforming growth factor, beta 1 | 7040 | TGFB1_HUMAN | |
| 92 | ERCC6 | excision repair cross-complementation group 6 | 2074 | ERCC6_HUMAN | |
| 93 | STK11 | serine/threonine kinase 11 | 6794 | STK11_HUMAN | |
| 94 | EP300 | E1A binding protein p300 | 2033 | EP300_HUMAN | |
| 95 | APTX | aprataxin | 54840 | APTX_HUMAN | |
| 96 | PML | promyelocytic leukemia | 5371 | PML_HUMAN | |
| 97 | GSK3B | glycogen synthase kinase 3 beta | 2932 | GSK3B_HUMAN | |
| 98 | HTT | huntingtin | 3064 | HD_HUMAN | |
| 99 | PRKCA | protein kinase C, alpha | 5578 | KPCA_HUMAN | |
| 100 | SSTR3 | somatostatin receptor 3 | 6753 | SSR3_HUMAN | |
| 101 | HELLS | helicase, lymphoid-specific | 3070 | HELLS_HUMAN | |
| 102 | APOC3 | apolipoprotein C-III | 345 | APOC3_HUMAN | |
| 103 | EEF2 | eukaryotic translation elongation factor 2 | 1938 | EF2_HUMAN | |
| 104 | ERCC3 | excision repair cross-complementation group 3 | 2071 | ERCC3_HUMAN | |
| 105 | TERF1 | telomeric repeat binding factor (NIMA-interacting) 1 | 7013 | TERF1_HUMAN | |
| 106 | PRKDC | protein kinase, DNA-activated, catalytic polypeptide | 5591 | PRKDC_HUMAN | |
| 107 | CAT | catalase | 847 | CATA_HUMAN | |
| 109 | ERCC5 | excision repair cross-complementation group 5 | 2073 | ERCC5_HUMAN | |
| 110 | AR | androgen receptor | 367 | ANDR_HUMAN | |
| 111 | GTF2H2 | general transcription factor IIH, polypeptide 2, 44kDa | 2966 | TF2H2_HUMAN | |
| 112 | XRCC5 | X-ray repair complementing defective repair in Chinese hamster cells 5 (double-strand-break rejoining) | 7520 | XRCC5_HUMAN | |
| 113 | PCNA | proliferating cell nuclear antigen | 5111 | PCNA_HUMAN | |
| 114 | FEN1 | flap structure-specific endonuclease 1 | 2237 | FEN1_HUMAN | |
| 115 | FAS | Fas cell surface death receptor | 355 | TNR6_HUMAN | |
| 116 | TERF2 | telomeric repeat binding factor 2 | 7014 | TERF2_HUMAN | |
| 117 | XRCC6 | X-ray repair complementing defective repair in Chinese hamster cells 6 | 2547 | KU70_HUMAN | |
| 118 | POLD1 | polymerase (DNA directed), delta 1, catalytic subunit | 5424 | DPOD1_HUMAN | |
| 119 | BAX | BCL2-associated X protein | 581 | BAX_HUMAN | |
| 120 | RB1 | retinoblastoma 1 | 5925 | RB_HUMAN | |
| 121 | EMD | emerin | 2010 | EMD_HUMAN | |
| 122 | GRB2 | growth factor receptor-bound protein 2 | 2885 | GRB2_HUMAN | |
| 123 | FOXO3 | forkhead box O3 | 2309 | FOXO3_HUMAN | |
| 124 | FOXO1 | forkhead box O1 | 2308 | FOXO1_HUMAN | |
| 125 | HSF1 | heat shock transcription factor 1 | 3297 | HSF1_HUMAN | |
| 126 | XPA | xeroderma pigmentosum, complementation group A | 7507 | XPA_HUMAN | |
| 127 | MSRA | methionine sulfoxide reductase A | 4482 | MSRA_HUMAN | |
| 128 | RECQL4 | RecQ helicase-like 4 | 9401 | RECQ4_HUMAN | |
| 129 | SOD2 | superoxide dismutase 2, mitochondrial | 6648 | SODM_HUMAN | |
| 130 | SOD1 | superoxide dismutase 1, soluble | 6647 | SODC_HUMAN | |
| 131 | FOXM1 | forkhead box M1 | 2305 | FOXM1_HUMAN | |
| 132 | COQ7 | coenzyme Q7 homolog, ubiquinone (yeast) | 10229 | COQ7_HUMAN | |
| 133 | CACNA1A | calcium channel, voltage-dependent, P/Q type, alpha 1A subunit | 773 | CAC1A_HUMAN | |
| 134 | LRP2 | low density lipoprotein receptor-related protein 2 | 4036 | LRP2_HUMAN | |
| 135 | AIFM1 | apoptosis-inducing factor, mitochondrion-associated, 1 | 9131 | AIFM1_HUMAN | |
| 136 | UCHL1 | ubiquitin carboxyl-terminal esterase L1 (ubiquitin thiolesterase) | 7345 | UCHL1_HUMAN | |
| 137 | APP | amyloid beta (A4) precursor protein | 351 | A4_HUMAN | |
| 138 | APOE | apolipoprotein E | 348 | APOE_HUMAN | |
| 139 | A2M | alpha-2-macroglobulin | 2 | A2MG_HUMAN | |
| 140 | SNCG | synuclein, gamma (breast cancer-specific protein 1) | 6623 | SYUG_HUMAN | |
| 141 | PRDX1 | peroxiredoxin 1 | 5052 | PRDX1_HUMAN | |
| 142 | PON1 | paraoxonase 1 | 5444 | PON1_HUMAN | |
| 143 | RELA | v-rel avian reticuloendotheliosis viral oncogene homolog A | 5970 | TF65_HUMAN | |
| 144 | IL6 | interleukin 6 | 3569 | IL6_HUMAN | |
| 145 | RGN | regucalcin | 9104 | RGN_HUMAN | |
| 146 | ATP5O | ATP synthase, H+ transporting, mitochondrial F1 complex, O subunit | 539 | ATPO_HUMAN | |
| 147 | RAD52 | RAD52 homolog, DNA repair protein | 5893 | RAD52_HUMAN | |
| 148 | TOP3B | topoisomerase (DNA) III beta | 8940 | TOP3B_HUMAN | |
| 149 | ERCC1 | excision repair cross-complementation group 1 | 2067 | ERCC1_HUMAN | |
| 150 | SIRT1 | sirtuin 1 | 23411 | SIR1_HUMAN | |
| 151 | HDAC1 | histone deacetylase 1 | 3065 | HDAC1_HUMAN | |
| 152 | HSPA9 | heat shock 70kDa protein 9 (mortalin) | 3313 | GRP75_HUMAN | |
| 153 | GPX1 | glutathione peroxidase 1 | 2876 | GPX1_HUMAN | |
| 154 | GSR | glutathione reductase | 2936 | GSHR_HUMAN | |
| 155 | GSS | glutathione synthetase | 2937 | GSHB_HUMAN | |
| 156 | GSTA4 | glutathione S-transferase alpha 4 | 2941 | GSTA4_HUMAN | |
| 157 | GSTP1 | glutathione S-transferase pi 1 | 2950 | GSTP1_HUMAN | |
| 158 | MT-CO1 | mitochondrially encoded cytochrome c oxidase I | 4512 | COX1_HUMAN | |
| 159 | HSPD1 | heat shock 60kDa protein 1 (chaperonin) | 3329 | CH60_HUMAN | |
| 160 | HSPA1A | heat shock 70kDa protein 1A | 3303 | HS71A_HUMAN | |
| 161 | HSPA1B | heat shock 70kDa protein 1B | 3304 | HSPA1B | |
| 162 | PCMT1 | protein-L-isoaspartate (D-aspartate) O-methyltransferase | 5110 | PIMT_HUMAN | |
| 163 | MAPK8 | mitogen-activated protein kinase 8 | 5599 | MK08_HUMAN | |
| 164 | YWHAZ | tyrosine 3-monooxygenase/tryptophan 5-monooxygenase activation protein, zeta | 7534 | 1433Z_HUMAN | |
| 165 | PTK2B | protein tyrosine kinase 2 beta | 2185 | FAK2_HUMAN | |
| 166 | PTK2 | protein tyrosine kinase 2 | 5747 | FAK1_HUMAN | |
| 167 | IL7 | interleukin 7 | 3574 | IL7_HUMAN | |
| 168 | MAPK14 | mitogen-activated protein kinase 14 | 1432 | MK14_HUMAN | |
| 169 | FGFR1 | fibroblast growth factor receptor 1 | 2260 | FGFR1_HUMAN | |
| 170 | SP1 | Sp1 transcription factor | 6667 | SP1_HUMAN | |
| 171 | FLT1 | fms-related tyrosine kinase 1 | 2321 | VGFR1_HUMAN | |
| 172 | JUN | jun proto-oncogene | 3725 | JUN_HUMAN | |
| 173 | MED1 | mediator complex subunit 1 | 5469 | MED1_HUMAN | |
| 174 | MAPK9 | mitogen-activated protein kinase 9 | 5601 | MK09_HUMAN | |
| 175 | MAPK3 | mitogen-activated protein kinase 3 | 5595 | MK03_HUMAN | |
| 176 | HMGB1 | high mobility group box 1 | 3146 | HMGB1_HUMAN | |
| 177 | CCNA2 | cyclin A2 | 890 | CCNA2_HUMAN | |
| 178 | HMGB2 | high mobility group box 2 | 3148 | HMGB2_HUMAN | |
| 179 | MAP3K5 | mitogen-activated protein kinase kinase kinase 5 | 4217 | M3K5_HUMAN | |
| 180 | TAF1 | TAF1 RNA polymerase II, TATA box binding protein (TBP)-associated factor, 250kDa | 6872 | TAF1_HUMAN | |
| 181 | LMNB1 | lamin B1 | 4001 | LMNB1_HUMAN | |
| 182 | SDHC | succinate dehydrogenase complex, subunit C, integral membrane protein, 15kDa | 6391 | C560_HUMAN | |
| 183 | FOXO4 | forkhead box O4 | 4303 | FOXO4_HUMAN | |
| 184 | HESX1 | HESX homeobox 1 | 8820 | HESX1_HUMAN | |
| 185 | PIK3R1 | phosphoinositide-3-kinase, regulatory subunit 1 (alpha) | 5295 | P85A_HUMAN | |
| 186 | BSCL2 | Berardinelli-Seip congenital lipodystrophy 2 (seipin) | 26580 | BSCL2_HUMAN | |
| 187 | AGPAT2 | 1-acylglycerol-3-phosphate O-acyltransferase 2 | 10555 | PLCB_HUMAN | |
| 188 | BMI1 | BMI1 proto-oncogene, polycomb ring finger | 648 | BMI1_HUMAN | |
| 189 | EEF1A1 | eukaryotic translation elongation factor 1 alpha 1 | 1915 | EF1A1_HUMAN | |
| 190 | TFAP2A | transcription factor AP-2 alpha (activating enhancer binding protein 2 alpha) | 7020 | AP2A_HUMAN | |
| 191 | BDNF | brain-derived neurotrophic factor | 627 | BDNF_HUMAN | |
| 192 | CREB1 | cAMP responsive element binding protein 1 | 1385 | CREB1_HUMAN | |
| 193 | ATF2 | activating transcription factor 2 | 1386 | ATF2_HUMAN | |
| 194 | TBP | TATA box binding protein | 6908 | TBP_HUMAN | |
| 195 | APEX1 | APEX nuclease (multifunctional DNA repair enzyme) 1 | 328 | APEX1_HUMAN | |
| 196 | HBP1 | HMG-box transcription factor 1 | 26959 | HBP1_HUMAN | |
| 197 | BUB1B | BUB1 mitotic checkpoint serine/threonine kinase B | 701 | BUB1B_HUMAN | |
| 198 | PTGS2 | prostaglandin-endoperoxide synthase 2 (prostaglandin G/H synthase and cyclooxygenase) | 5743 | PGH2_HUMAN | |
| 199 | HSPA8 | heat shock 70kDa protein 8 | 3312 | HSP7C_HUMAN | |
| 200 | SIN3A | SIN3 transcription regulator family member A | 25942 | SIN3A_HUMAN | |
| 201 | CDK1 | cyclin-dependent kinase 1 | 983 | CDK1_HUMAN | |
| 202 | TFDP1 | transcription factor Dp-1 | 7027 | TFDP1_HUMAN | |
| 203 | DDIT3 | DNA-damage-inducible transcript 3 | 1649 | DDIT3_HUMAN | |
| 204 | POLA1 | polymerase (DNA directed), alpha 1, catalytic subunit | 5422 | DPOLA_HUMAN | |
| 205 | MAPT | microtubule-associated protein tau | 4137 | TAU_HUMAN | |
| 206 | CTGF | connective tissue growth factor | 1490 | CTGF_HUMAN | |
| 207 | HDAC2 | histone deacetylase 2 | 3066 | HDAC2_HUMAN | |
| 208 | MAX | MYC associated factor X | 4149 | MAX_HUMAN | |
| 209 | MXD1 | MAX dimerization protein 1 | 4084 | MAD1_HUMAN | |
| 210 | MDM2 | MDM2 proto-oncogene, E3 ubiquitin protein ligase | 4193 | MDM2_HUMAN | |
| 211 | SUMO1 | small ubiquitin-like modifier 1 | 7341 | SUMO1_HUMAN | |
| 212 | H2AFX | H2A histone family, member X | 3014 | H2AX_HUMAN | |
| 213 | HOXB7 | homeobox B7 | 3217 | HXB7_HUMAN | |
| 214 | HOXC4 | homeobox C4 | 3221 | HXC4_HUMAN | |
| 215 | JAK2 | Janus kinase 2 | 3717 | JAK2_HUMAN | |
| 216 | ESR1 | estrogen receptor 1 | 2099 | ESR1_HUMAN | |
| 217 | LEP | leptin | 3952 | LEP_HUMAN | |
| 218 | LEPR | leptin receptor | 3953 | LEPR_HUMAN | |
| 219 | NFKBIA | nuclear factor of kappa light polypeptide gene enhancer in B-cells inhibitor, alpha | 4792 | IKBA_HUMAN | |
| 220 | CLU | clusterin | 1191 | CLUS_HUMAN | |
| 221 | MTOR | mechanistic target of rapamycin (serine/threonine kinase) | 2475 | MTOR_HUMAN | |
| 222 | GHRHR | growth hormone releasing hormone receptor | 2692 | GHRHR_HUMAN | |
| 223 | CTNNB1 | catenin (cadherin-associated protein), beta 1, 88kDa | 1499 | CTNB1_HUMAN | |
| 224 | PSEN1 | presenilin 1 | 5663 | PSN1_HUMAN | |
| 225 | DLL3 | delta-like 3 (Drosophila) | 10683 | DLL3_HUMAN | |
| 226 | CDKN2A | cyclin-dependent kinase inhibitor 2A | 1029 | CDN2A_HUMAN | |
| 227 | PPP1CA | protein phosphatase 1, catalytic subunit, alpha isozyme | 5499 | PP1A_HUMAN | |
| 228 | DBN1 | drebrin 1 | 1627 | DREB_HUMAN | |
| 229 | NOG | noggin | 9241 | NOGG_HUMAN | |
| 230 | ELN | elastin | 2006 | ELN_HUMAN | |
| 231 | ATR | ATR serine/threonine kinase | 545 | ATR_HUMAN | |
| 232 | UCP3 | uncoupling protein 3 (mitochondrial, proton carrier) | 7352 | UCP3_HUMAN | |
| 233 | ZMPSTE24 | zinc metallopeptidase STE24 | 10269 | FACE1_HUMAN | |
| 234 | TP63 | tumor protein p63 | 8626 | P63_HUMAN | |
| 235 | UCP2 | uncoupling protein 2 (mitochondrial, proton carrier) | 7351 | UCP2_HUMAN | |
| 236 | POLB | polymerase (DNA directed), beta | 5423 | DPOLB_HUMAN | |
| 237 | GCLC | glutamate-cysteine ligase, catalytic subunit | 2729 | GSH1_HUMAN | |
| 238 | GCLM | glutamate-cysteine ligase, modifier subunit | 2730 | GSH0_HUMAN | |
| 239 | SIRT6 | sirtuin 6 | 51548 | SIRT6_HUMAN | |
| 240 | BUB3 | BUB3 mitotic checkpoint protein | 9184 | BUB3_HUMAN | |
| 241 | RAE1 | ribonucleic acid export 1 | 8480 | RAE1L_HUMAN | |
| 242 | PMCH | pro-melanin-concentrating hormone | 5367 | MCH_HUMAN | |
| 243 | MLH1 | mutL homolog 1 | 4292 | MLH1_HUMAN | |
| 244 | CSNK1E | casein kinase 1, epsilon | 1454 | KC1E_HUMAN | |
| 245 | STUB1 | STIP1 homology and U-box containing protein 1, E3 ubiquitin protein ligase | 10273 | CHIP_HUMAN | |
| 246 | PPM1D | protein phosphatase, Mg2+/Mn2+ dependent, 1D | 8493 | PPM1D_HUMAN | |
| 247 | CHEK2 | checkpoint kinase 2 | 11200 | CHK2_HUMAN | |
| 248 | PCK1 | phosphoenolpyruvate carboxykinase 1 (soluble) | 5105 | PCKGC_HUMAN | |
| 249 | ARHGAP1 | Rho GTPase activating protein 1 | 392 | RHG01_HUMAN | |
| 250 | CDC42 | cell division cycle 42 | 998 | CDC42_HUMAN | |
| 251 | ARNTL | aryl hydrocarbon receptor nuclear translocator-like | 406 | BMAL1_HUMAN | |
| 252 | CLOCK | clock circadian regulator | 9575 | CLOCK_HUMAN | |
| 253 | HIC1 | hypermethylated in cancer 1 | 3090 | HIC1_HUMAN | |
| 254 | PAPPA | pregnancy-associated plasma protein A, pappalysin 1 | 5069 | PAPP1_HUMAN | |
| 255 | ADCY5 | adenylate cyclase 5 | 111 | ADCY5_HUMAN | |
| 256 | PPARGC1A | peroxisome proliferator-activated receptor gamma, coactivator 1 alpha | 10891 | PRGC1_HUMAN | |
| 257 | GPX4 | glutathione peroxidase 4 | 2879 | GPX4_HUMAN | |
| 258 | UCP1 | uncoupling protein 1 (mitochondrial, proton carrier) | 7350 | UCP1_HUMAN | |
| 259 | FGF23 | fibroblast growth factor 23 | 8074 | FGF23_HUMAN | |
| 260 | EFEMP1 | EGF containing fibulin-like extracellular matrix protein 1 | 2202 | FBLN3_HUMAN | |
| 261 | ERCC4 | excision repair cross-complementation group 4 | 2072 | XPF_HUMAN | |
| 262 | CETP | cholesteryl ester transfer protein, plasma | 1071 | CETP_HUMAN | |
| 263 | PPARG | peroxisome proliferator-activated receptor gamma | 5468 | PPARG_HUMAN | |
| 264 | AGTR1 | angiotensin II receptor, type 1 | 185 | AGTR1_HUMAN | |
| 265 | CISD2 | CDGSH iron sulfur domain 2 | 493856 | CISD2_HUMAN | |
| 266 | EEF1E1 | eukaryotic translation elongation factor 1 epsilon 1 | 9521 | MCA3_HUMAN | |
| 267 | EPS8 | epidermal growth factor receptor pathway substrate 8 | 2059 | ES8L2_HUMAN | |
| 268 | KCNA3 | potassium channel, voltage gated shaker related subfamily A, member 3 | 3738 | KCNA3_HUMAN | |
| 269 | SIRT7 | sirtuin 7 | 51547 | SIR7_HUMAN | |
| 270 | SLC13A1 | solute carrier family 13 (sodium/sulfate symporter), member 1 | 6561 | A4D0X1_HUMAN | |
| 271 | SOCS2 | suppressor of cytokine signaling 2 | 8835 | SOCS2_HUMAN | |
| 273 | TPP2 | tripeptidyl peptidase II | 7174 | TPP2_HUMAN | |
| 274 | TP53BP1 | tumor protein p53 binding protein 1 | 7158 | TP53B_HUMAN | |
| 275 | SIRT3 | sirtuin 3 | 23410 | SIR3_HUMAN | |
| 276 | NCOR2 | nuclear receptor corepressor 2 | 9612 | NCOR2_HUMAN | |
| 277 | SUN1 | Sad1 and UNC84 domain containing 1 | 23353 | SUN1_HUMAN | |
| 278 | BAK1 | BCL2-antagonist/killer 1 | 578 | BAK_HUMAN | |
| 279 | IGFBP2 | insulin-like growth factor binding protein 2, 36kDa | 3485 | IBP2_HUMAN | |
| 280 | PYCR1 | pyrroline-5-carboxylate reductase 1 | 5831 | P5CR1_HUMAN | |
| 281 | TP73 | tumor protein p73 | 7161 | P73_HUMAN | |
| 282 | CNR1 | cannabinoid receptor 1 (brain) | 1268 | CNR1_HUMAN | |
| 283 | NFE2L2 | nuclear factor, erythroid 2-like 2 | 4780 | NF2L2_HUMAN | |
| 284 | CDKN1A | cyclin-dependent kinase inhibitor 1A (p21, Cip1) | 1026 | CDN1A_HUMAN | |
| 285 | PDGFRA | platelet-derived growth factor receptor, alpha polypeptide | 5156 | PGFRA_HUMAN | |
| 286 | PIK3CA | phosphatidylinositol-4,5-bisphosphate 3-kinase, catalytic subunit alpha | 5290 | PK3CA_HUMAN | |
| 287 | C1QA | complement component 1, q subcomponent, A chain | 712 | C1QA_HUMAN | |
| 288 | CDKN2B | cyclin-dependent kinase inhibitor 2B (p15, inhibits CDK4) | 1030 | CDN2B_HUMAN | |
| 289 | EIF5A2 | eukaryotic translation initiation factor 5A2 | 56648 | IF5A2_HUMAN | |
| 290 | MIF | macrophage migration inhibitory factor (glycosylation-inhibiting factor) | 4282 | MIF_HUMAN | |
| 291 | DGAT1 | diacylglycerol O-acyltransferase 1 | 8694 | DGAT1_HUMAN | |
| 292 | MT1E | metallothionein 1E | 4493 | MT1E_HUMAN | |
| 293 | FGF21 | fibroblast growth factor 21 | 26291 | FGF21_HUMAN | |
| 294 | HTRA2 | HtrA serine peptidase 2 | 27429 | HTRA2_HUMAN | |
| 295 | GSK3A | glycogen synthase kinase 3 alpha | 2931 | GSK3A_HUMAN | |
| 296 | NUDT1 | nudix (nucleoside diphosphate linked moiety X)-type motif 1 | 4521 | 8ODP_HUMAN | |
| 297 | IKBKB | inhibitor of kappa light polypeptide gene enhancer in B-cells, kinase beta | 3551 | IKKB_HUMAN | |
| 298 | SQSTM1 | sequestosome 1 | 8878 | SQSTM_HUMAN | |
| 299 | CDK7 | cyclin-dependent kinase 7 | 1022 | CDK7_HUMAN | |
| 300 | GRN | granulin | 2896 | GRN_HUMAN | |
| 301 | SERPINE1 | serpin peptidase inhibitor, clade E (nexin, plasminogen activator inhibitor type 1), member 1 | 5054 | PAI1_HUMAN | |
| 302 | SPRTN | SprT-like N-terminal domain | 83932 | SPRTN_HUMAN | |
| 303 | RICTOR | RPTOR independent companion of MTOR, complex 2 | 253260 | RICTR_HUMAN | |
| 304 | CTF1 | cardiotrophin 1 | 1489 | CTF1_HUMAN | |
| 305 | TRAP1 | TNF receptor-associated protein 1 | 10131 | TRAP1_HUMAN | |
| 306 | TRPV1 | transient receptor potential cation channel subfamily V member 1 | 7442 | TRPV1_HUMAN | |
| 307 | NFE2L1 | nuclear factor, erythroid 2-like 1 | 4779 | NF2L1_HUMAN | |
| 308 | IFNB1 | Interferon beta | 3456 | IFNB_HUMAN | |
| 309 | GDF11 | growth differentiation factor 11 | 10220 | A0A024RB20_HUMAN | |
